# A Bioinspired Mastoparan Exhibits Concentration-Dependent Anti-Bacterial Activity via Membrane Disruption

**DOI:** 10.1021/acsami.5c14290

**Published:** 2025-11-24

**Authors:** Gisele R. Rodrigues, Marco Fornasier, Lucrezia Caselli, Martin Malmsten, Emma Sparr, Peter Jönsson, Octavio L. Franco

**Affiliations:** † Centro de Análises Proteômicas e Bioquímicas, Pós-Graduação em Ciências Genômicas e Biotecnologia, Universidade Católica de Brasília, Brasília, Distrito Federal 70790160, Brazil; ‡ Division of Physical Chemistry, 5193 Lund University, Lund SE-221 00, Sweden; § S-inova Biotech, Programa de Pós-Graduação em Biotecnologia, Universidade Católica Dom Bosco, Campo Grande, Mato Grosso do Sul 79117900, Brazil; ∥ Department of Pharmacy, University of Copenhagen, Copenhagen DK-2100, Denmark

**Keywords:** antimicrobial peptides, bacteria, lipid membranes, mastoparans, membranolytic effect

## Abstract

Antimicrobial peptides are widely investigated in the literature, but their mechanism of action and effects on lipid membranes are not completely understood from a physicochemical perspective. In this study, we employed a bioinspired mastoparan from wasp venom, mast-MO, and characterized its interactions with model lipid membranes, either as a supported lipid bilayer or as free-standing vesicles in solution. An array of complementary physicochemical characterization techniques was employed to study the surface activity of the peptide alone and how its adsorption affects lipid membrane properties in terms of lateral organization and integrity. We found that peptide action is related to its intrinsic surface activity, resulting in disrupted lipid packing of supported membranes and vesicles via a concentration-dependent mechanism. Changing solution conditions, e.g., ionic strength and pH, altered the electrostatic interactions between the membrane and mast-MO, resulting in less significant adsorption. This mechanism of action was also validated *in vitro* for Gram-negative *E. coli* bacteria, demonstrating rapid action (within 15 min) and potent antimicrobial activity. These results provide new information on the molecular effects of mastoparan’s interactions with membranes.

## Graphical abstract

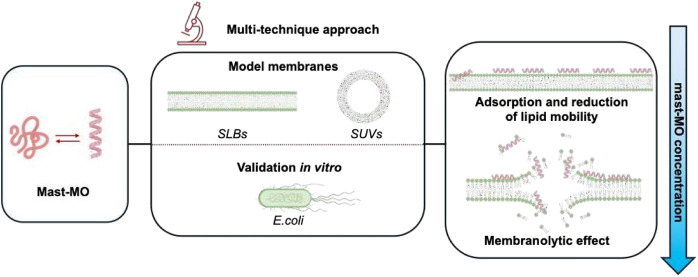

## Introduction

The rise in multidrug-resistant bacteria represents an alarming global health issue.^1^ Antibiotic-resistant infections are predicted to cause 10 million deaths annually by 2050 if new antimicrobial strategies are not developed and other measures taken to reduce unnecessary use of antibiotics, as well as antibiotic spread.^1^ Similarly, increased attention is currently being devoted to utilizing natural materials as a source of novel approaches to combat bacterial infections. Among them, anti-microbial peptides (AMPs), produced by nearly all organisms, present a promising alternative to conventional antibiotics.^2^ These short amphiphiles display potent activity against micro-organisms, including different bacterial strains.^3,4^ Due to their positive charge under physiological conditions, such peptides can bind to bacterial membranes, which are typically negatively charged^5,6^ or to membrane domains with a high local anionic charge density.^7–9^ The mechanism of action of many AMPs is still under debate. However, they may interact with microbial membranes via several pathways, which can be summarized as (i) partitioning into the lipid membrane, permeating through it, and exerting a cytotoxic effect inside the cell, or (ii) adsorbing on the lipid membrane and inducing a perturbation in the lipid packing (e.g., alterations in the local curvature of the bilayer, lateral segregation, or asymmetry between the lipid leaflets). All these changes may subsequently lead to membrane disruption.^10,11^ Elucidating all these molecular phenomena is not trivial, as the same peptide may present a concentration-dependent behavior.^12^

Several AMPs have been enlisted and studied to tackle antibiotic resistant infections. Here, we focus on the class of mastoparan peptides, due to their potent *in vitro* action at low concentrations. These small peptides, which have a positive net charge at physiological pH, are initially isolated from wasp venoms. They typically adopt an α-helical structure upon binding to negatively charged interfaces as microbial membranes.^13^ Depending on their amino-acid sequence, mastoparans may act via multiple mechanisms against microorganisms, e.g., membrane adsorption, further dissolution of lipids in lipid-peptide coassembly with membrane disruption, and interaction with intracellular targets.^14,15^ Through this, they exhibit broad-spectrum antimicrobial activity, rapid antimicrobial effects, and immunomodulatory properties.^14,15^ Their structural flexibility, thereby enabling them to overcome the numerous defense mechanisms employed by microorganisms to evade antimicrobial agents.^15^

Mastoparans have demonstrated diverse biological activities, including antimicrobial, cytotoxic, and immunomodulatory effects.^16^ Mastoparan-MO, also named Mast-MO, is a synthetic analog of the natural mastoparan peptide, engineered to enhance antimicrobial, antiviral, and immunomodulatory properties while reducing cytotoxicity.^17,18^ As determined by NMR, mast-MO adopts an α-helical structure upon binding to bacterial membranes and exhibits enhanced antibacterial properties comparable to standard-of-care antibiotics *in vitro* and *in vivo*.^17^ The authors suggested that the mechanism of action of mast-MO and some derivatives involves rapidly binding and perturbing the lipid packing. The studies focused on mast-MO and its derivatives mainly employed spectroscopies such as circular dichroism^19^ or surface plasmon resonance^14^ coupled with *in vitro* and *in vivo* assays. Findings based on these approaches suggested a detergent-like mechanism of action, resulting from a strong interaction with lipid membranes and subsequent bilayer disruption. Nevertheless, there is still a lack of knowledge on the effect of the peptide on the nano- and mesoscale arrangement of the lipid membrane. Specifically, the interactions of AMPs with lipid membranes are crucial points in evaluating the mechanism of action, particularly in terms of lateral organization and membrane remodeling.

Here, we focused on the physicochemical characterization of mast-MO (FLPIIINLKALAALAKKIL) and its adsorption to model membranes and bacteria. The present investigations aimed to address this by characterizing: (i) the mechanism of action of mast-MO at the nano- and mesoscale and (ii) the effect of the peptide on model lipid membranes and then confirm such investigations in suspensions of *E. coli*. Our results showed that mast-MO has a concentration-dependent action on model membranes, which is influenced by solution conditions (e.g., pH and ionic strength), inferred as a consequence of perturbing the lipid membrane with subsequent disruption and aggregation of the lipids. The potent activity was also tested on *E. coli* using a dead-alive assay, demonstrating that a low concentration is sufficient to induce aggregation and cell death in this bacterial strain.

## Results

### Adsorption of the Peptide to Bare Surfaces and Supported Lipid Bilayers (SLBs)

Previous studies have suggested that mast-MO has a membrane disrupting action on lipid membranes;^14,17^ however, there is still a lack of understanding of the surface activity of the peptide and how this is linked to its mechanism of action. For this reason, quartz crystal microbalance with dissipation (QCM-D) measurements were performed to investigate peptide adsorption on either bare silica surfaces (hydrophilic) or silica surfaces functionalized with trimethyloctylsilane (hydrophobic).^25^ Overall, QCM-D reports on the amount of ma.ss adsorbed (coupled with water) in terms of frequency shift, *Δf*, of an oscillating crystal, and the decay of this oscillation over time is connected to the dissipation, *ΔD*, of the system, which can inform on the viscoelasticity of the layer. Figure 1 summarizes the results of the experiments of mast-MO in HBS buffer (pH 7.4, I = 162 mM) on these hydrophilic (Figure 1A) and hydrophobic (Figure 1B) surfaces for concentrations ranging from 50 nM to 50 μM. The choice of using 50 μM as upper concentration limit is based on prior work of the group,^17^ which showed mast-MO is nontoxic up to 200 μM in vitro and in vivo, making it a safe and relevant reference for antimicrobial testing. For the hydrophobic surfaces, adsorption occurs gradually, reaching a stable level within 20 min after each addition of the peptide. There is no detectable adsorption to hydrophilic surfaces at the lower concentrations, while there is significant adsorption (corresponding to (−17.5 ± 0.5) Hz) at the highest peptide concentration investigated, 50 μM. Rinsing with HBS leads to a fast desorption of a fraction of the peptide from both surfaces, corresponding to a loss of ca. 20% after 30 min of rinsing. A second step of rinsing in pure water results in slower desorption, which does not reach a steady state within the time frame of the experiment. In conclusion, mast-MO adsorbs to both surfaces. This suggests that the AMP exhibits surface activity, with no specific preference for either hydrophilic or hydrophobic surfaces at this concentration regime. The dissipation, *ΔD*, increases by increasing the peptide concentration (from 50 nM to 50 μM) for both surfaces, highlighting a viscoelastic behavior of the adsorbed mast-MO layer (*ΔD* > 1 ppm).

**Figure 1:**
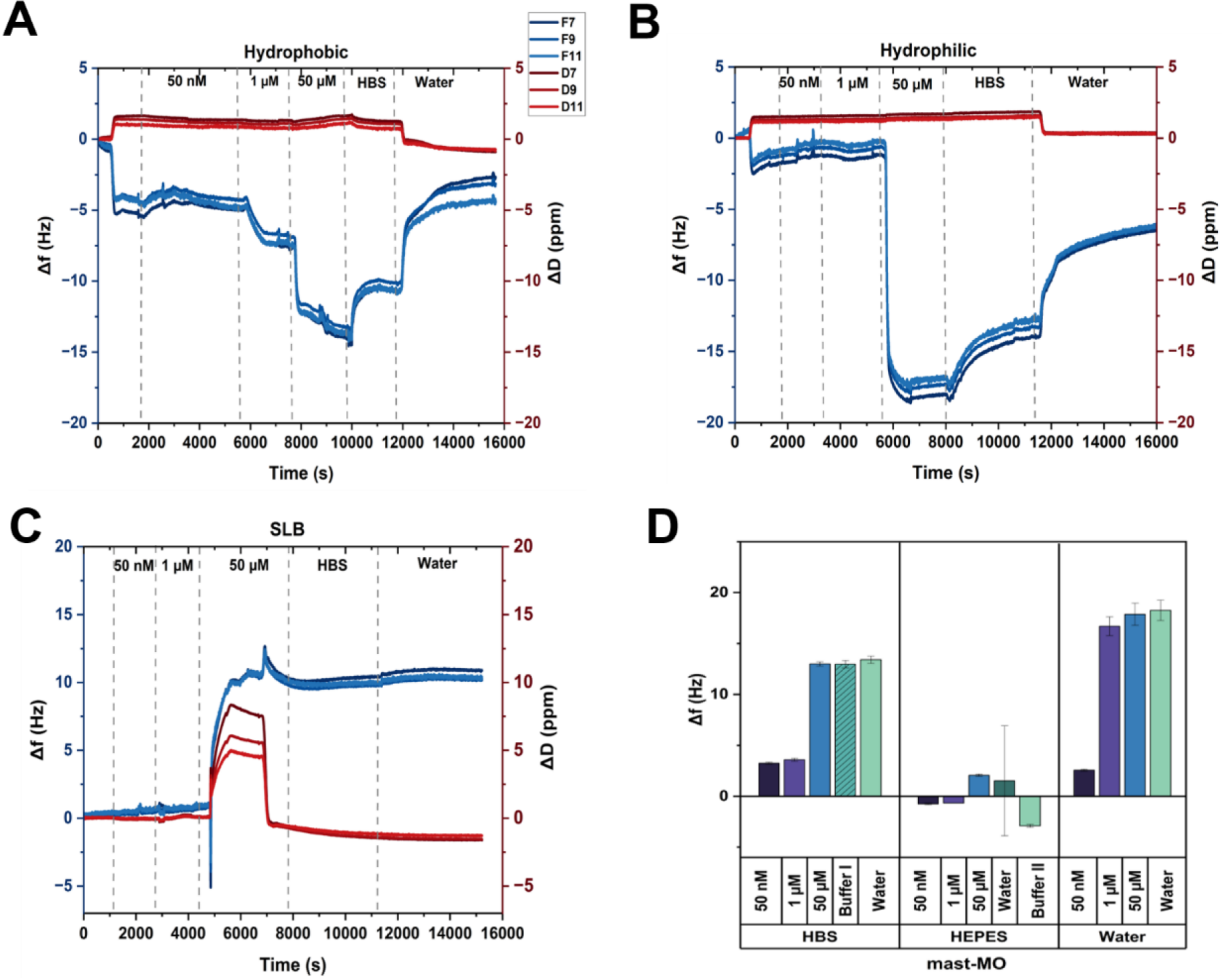
QCM-D raw data and analysis of mast-MO alone and in the presence of preformed SLBs. **A** and **B** show representative raw data from a QCM-D experiment on hydrophobic and hydrophilic surfaces, respectively, reporting the trend of the 7th, 9th, and 11th overtones as the concentration of the AMP increases from 0 to 50 μM, followed by rinsing with HBS and then water. **C** reports on the same kind of information for conditions when the QCM-D crystal is covered with a SLB of POPC: POPG (75:25) is formed on the surface of the QCM-D crystal. **D** shows the steady state values of the 9th overtone of *Δf* in each step for the different solution conditions under investigation.

Next, we evaluated the adsorption of mast-MO to SLBs composed of POPC: POPG (75:25, mol/mol %), a model mimic of bacterial membranes.^26^ These bilayers are negatively charged and are commonly used as a model membrane for how antimicrobials adsorb and perturb membranes.^7,27^ The membranes were formed onto silica substrates, resulting in SLBs with an average *Δf* of (−23 ± 1) Hz, as shown in Figure S1. After normalization, peptide adsorption to SLBs was plotted in Figure 1C and D for the three peptide concentrations under investigation. A slight change in both *Δf* and *ΔD* was observed at 50 nM and 1 μM of mast-MO, which may be related to lipid desorption from the sensor. Upon adding 50 μM peptide, the increase in *Δf* becomes more evident, accompanied by a spreading of the overtones of the dissipation signal. This means that the surface layer is viscoelastic, e.g., the adsorbed layer is coupled with water and protrudes into the aqueous bulk. The observed increase in *Δf* and *ΔD* can be attributed to membrane destabilization and disruption caused by the association of peptide and membrane components into assemblies in the bulk solution. Rinsing with buffer and water does not induce any further change in the recorded signals. To capture the effect of the adsorption on SLBs, we repeated the QCM-D experiments with lower ionic strength; HEPES buffer (pH 7.4, 1 mM of ionic strength, I) and in pure water (I = 0, pH not controlled), (see Figure SI2 for each measurement). Figure 1D summarizes the results of the experiments performed in these three different ionic strengths. Each of the bars of the histogram represents the steady state frequency shift of each step of the experiment, meaning (i) formation of the bilayer, (ii) addition of mast-MO at different concentrations, and (iii) rinsing in the desired medium. This experiment concludes that the removal of lipids due to mast-MO adsorption seems stronger in HBS and water, with little to no effect in the HEPES buffer.

### Effect of the Peptide on Membrane Fluidity and Integrity

To investigate the effect of the peptide adsorption on bilayer properties, total internal reflection fluorescence (TIRF) and fluorescence recovery after photobleaching (FRAP) measurements were performed on SLBs composed of POPC: POPG in HBS. As the peptide binds to anionic membranes, SLBs composed of only POPC as a negative control.

TIRF microscopy enables the investigation of surface phenomena by illuminating the sample only up to 200 nm from the glass slide surface. Additionally, FRAP provides information on the lateral diffusion of a fluorescent dye in the two-dimensional plane of the bilayer.

TIRF images were acquired before and after mast-MO addition to the SLBs and are presented in Figure 2A and B. Overall, the peptide adsorption does not significantly affect the homogeneity of the SLBs until the highest concentration of mast-MO is reached. In this case, the membrane is disrupted, as only membrane fragments (bright fluorescent spots with defined contours in Figure 2B) are observed floating out of the field of view and out of focus. The highest mast-MO concentration led to complete disruption of membrane organization at 50,000 nM. In this manner, it was not possible to perform a FRAP experiment, and this is shown both in Figure 2B and D. It was highlighted by fragments of membranes visible in the field of view, hinting at a lack of structural integrity of the SLB and, hence, to the membranolytic effect of mast-MO. The lateral diffusion of lipids within the membrane is already reduced at 50 nM mast-MO, as indicated by the FRAP measurements, which report on the values of the diffusion coefficient of the lipid probe (Figure 2C and D).

**Figure 2:**
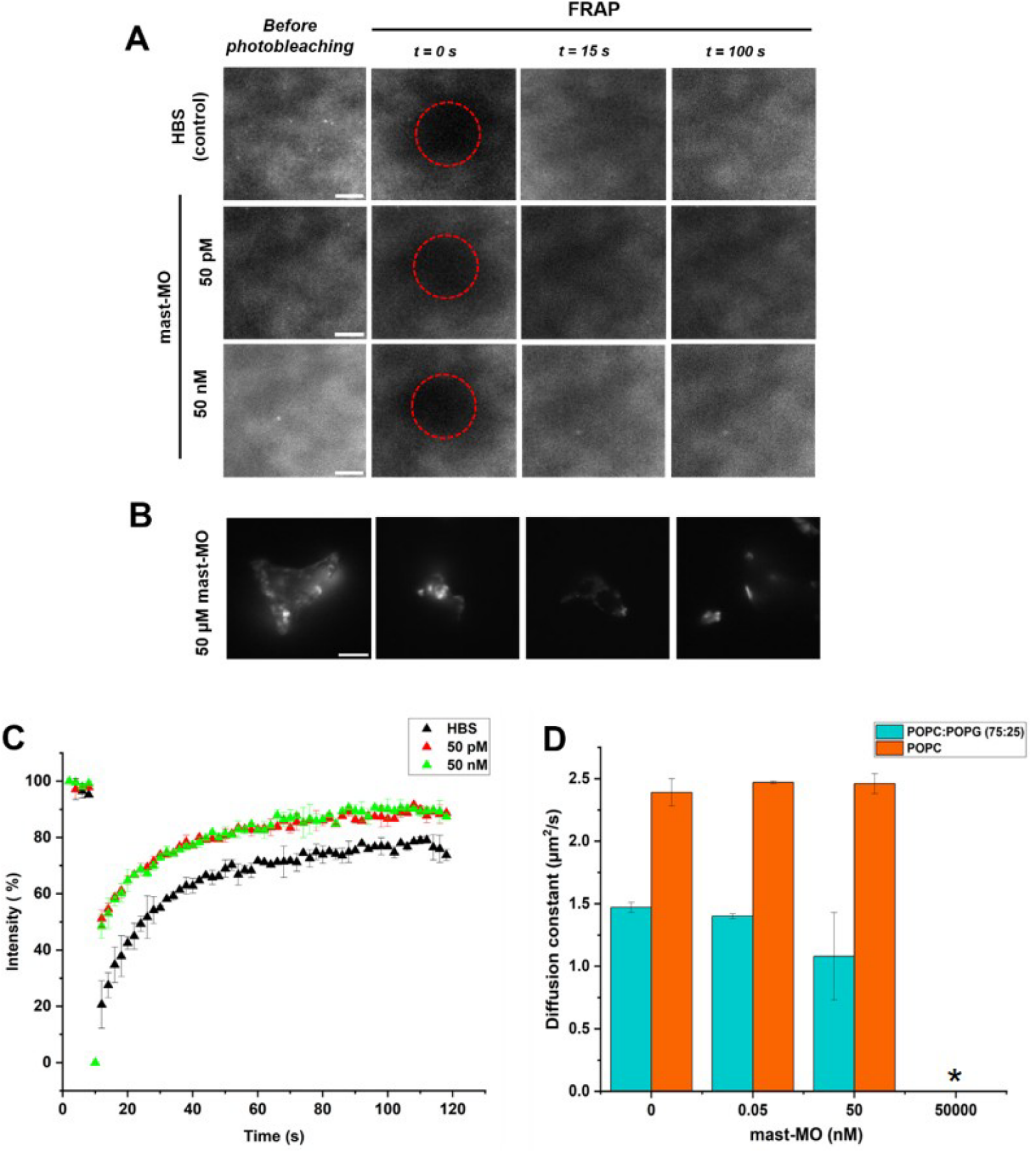
Fluorescence microscopy measurements on SLBs composed of POPC: POPG (75:25) in HBS. **A** and **B** show representative TIRF images acquired during the FRAP experiment after incubating the SLBs with different concentrations of mast-MO for 10 min. The red dotted circles represent the photobleached area of the FRAP experiment. **C** shows results on the normalized fluorescence intensity (%) over time evaluated within the red dotted region of **A** during a FRAP experiment for the before (HBS, black triangles) and after addition of the peptide (50 pM and 50 nM are red and green triangles, respectively). **D** show data from the panel C using the method from Jönsson et al.^21^ to analyze the diffusion coefficient, *D*, of the lipid-dye DHPE-OG as a function of mast-MO concentration, and * represents to complete disruption of membrane organization at 50,000 nM. The description of these colored images is referred to the online version of the manuscript. All measurements were performed in triplicate on independent samples to ensure statistical analysis, and the data are reported as average value ± SD. In all images, the scale bar corresponds to 5 μm.

In control experiments with POPC, a significant change in membrane fluidity was observed only at the highest concentration of mast-MO (Figures 2C and S3 for POPC: POPG and POPC only, respectively), due to peptide crowding at the membrane interface, indicating adsorption to zwitterionic membranes at high concentrations. This effect is not surprising, as it may be related to the induction of defects in the membrane rather than actual adsorption.

### Adsorption of Mast-MO to Small Unilamellar Vesicles: Effect on Size, Zeta Potential, and Morphology of SUVs

As peptide adsorption to SLBs may be affected by the underlying surface, SUVs with the same compositions as the ones used in the surface-sensitive experiments were prepared and measured via DLS and ELS after incubation with the peptide (Figure 3A and B). These experiments were performed for the same solution as used in the QCM-D measurements. First, ζ-potential measurements were carried out to evaluate whether surface potential changes due to peptide adsorption. For all solution conditions, the ζ-potential is virtually constant until a threshold concentration of mast-MO is reached. Above this, the ζ-potential increases by increasing the peptide concentration, eventually leading to reversed charge to positive ζ-potential values.

**Figure 3:**
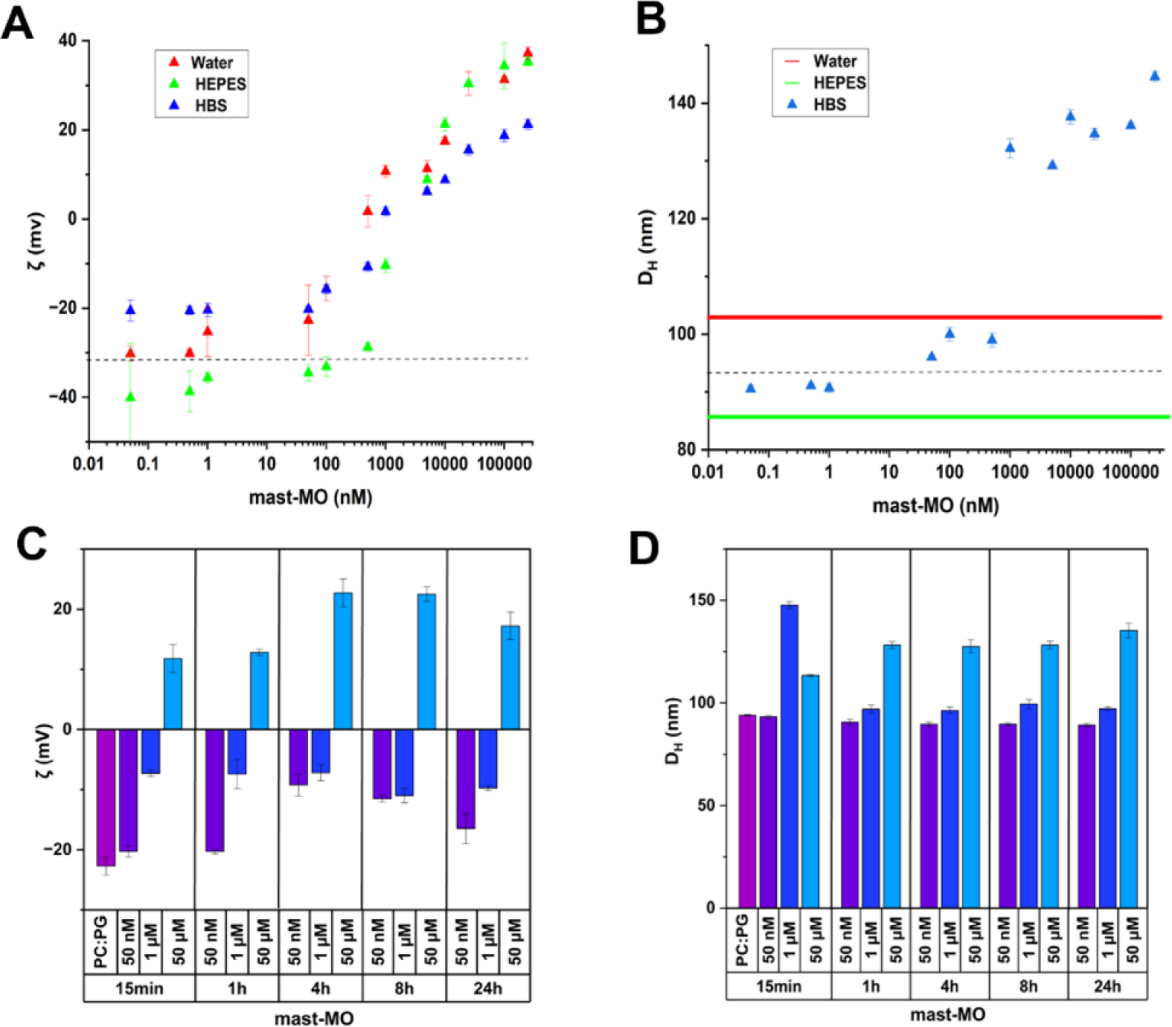
ELS and ELS results of SUVs composed by POPC: POPG upon addition of different aliquots of mast-MO in different media. **A** and **B** report the zeta potential and apparent hydrodynamic diameter, Dh, respectively, after incubation with mast-MO. The vesicles and mast-MO dilutions were prepared in pure water (red triangles), 10 mM HEPES pH 7.4 (green triangles), and 10 mM HBS pH 7.4 (blue triangles). In **B**, the red and green dotted lines correspond to the average sizes of POPC: POPG (75:25) vesicles after addition of the different peptide concentrations in water and HEPES, respectively, as no specific trend was observed (see Figure SI5). **C** and **D** show the measurements on three selected concentrations of mast-MO after incubation with POPC: POPG vesicles for 15 min, 1, 4, 8, and 24 h. All measurements were performed in triplicate, and the data are reported as average value ± SD.

The apparent hydrodynamic diameter of the SUVs prepared in HBS increased upon increasing mast-MO concentration. In contrast, the hydrodynamic diameter was constant for the other solution conditions. The observed increase in size at a peptide concentration of ca. 1 μM in HBS buffer correlates with the almost neutral ζ-potential observed in the ELS measurements. Similar trends of the ζ-potential were observed for vesicles composed of POPC upon peptide addition, while the apparent hydrodynamic diameter is constant, meaning that the peptide adsorbs but does not induce any size change in this concentration regime. Finally, DLS and ELS measurements were performed at different incubation times (15 min, 1, 4, 8, and 24 h) at the selected concentrations of 50 nM, 1 μM, and 50 μM of mast-MO in HBS, (Figure 3C and D) showing that changes in size and ζ-potential occur fast after peptide addition and that waiting for longer incubation times does not change the aggregate size or charge.

Cryo-TEM was employed to further study the morphology of SUVs upon adsorption of mast-MO. The micrographs of the SUVs without peptide (control) and after adding 50 nM, 1 μM, and 50 μM are reported in Figure 4. The conditions chosen for the cryo-TEM studies are the same as those used for the DLS and ELS measurements, facilitating comparison. In the absence of peptide, the SUVs are spherical with a low degree of multilamellarity (Figure 4A). When 50 nM of mast-MO was added, a slight increase in lamellarity (e.g., multilamellar vesicles) was observed (Figure 4B, top panel). Such a change in morphology becomes more evident at higher peptide concentration (1 μM); some vesicles are deformed in an ellipsoidal shape, and multivesicular vesicles start to appear, i.e., vesicles contained inside other vesicular objects, together with multilamellar ones, as shown in Figure 4B, medium panel. The highest concentration of mast-MO (50 μM) has the most dramatic effect on the shape and aggregation of the vesicles; overall, aggregation of lipids (indicated by blue arrows) and deformation of the SUVs (indicated by orange arrows) are observed (Figure 4D, lower panel).

**Figure 4:**
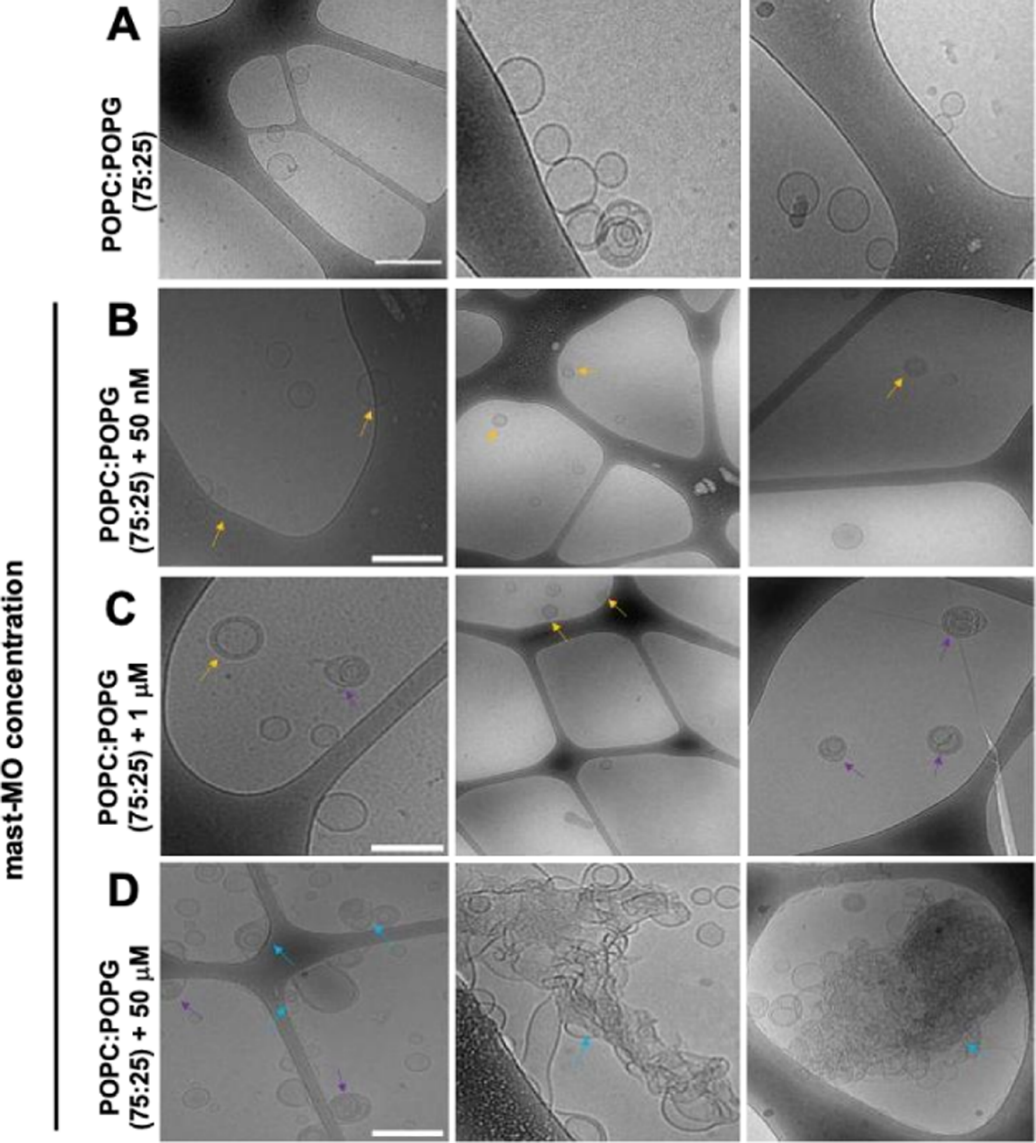
Effect of mast-MO on SUVs’ morphology studied with cryo-TEM. Micrographs of POPC: POPG (75:25) in HBS without peptide in **A**, after the addition of 50 nM in **B**, 1 μM in **C**, and 50 μM in **D** of mast-MO, and incubating the samples for 15 min at 25 °C. The yellow arrow points to the multilamellar vesicles, the purple arrow indicates the multivesicular vesicles, the cyan arrow indicates multilamellar vesicles, and the aggregation of vesicles. The scale bar corresponds to 200 nm in all images.

### Conformational Changes upon Binding to Small Unilamellar Vesicles (SUVs)

CD experiments were performed to assess the peptide conformation for solutions containing SUVs and a 5 μM peptide. These measurements were done in water only, as HEPES and chloride ions absorb light in the UV region, precluding structural analysis.

Samples containing mast-MO were then titrated with different amounts of SUVs, gradually increasing the vesicle concentration and covering lipid/peptide (L:P) ratios ranging from 1 to 200. The CD spectra of mast-MO for the free peptide and at the different L:P is presented in Figure SI4. The peptide showed a random coil conformation in the absence of vesicles. It then underwent a conformational change from random coil to α-helix upon binding to anionic membranes, consistent with previous studies in the literature.^28,29^ This transition is partial at L:P 1 but becomes more evident at higher vesicles concentration.

### Antibacterial Effects

Confocal microscopy experiments were performed to investigate the antimicrobial effects of mast-MO against *E. coli*, in order to prove the effects highlighted in model systems. A two-color fluorescence LIVE/DEAD assay was employed, enabling differentiation between bacteria with intact cell membranes (stained in green) and those with damaged membranes (stained in red) at different peptide concentrations at pH 7.4. Representative confocal microscopy images are shown in Figure 5A, while the percentages of live/dead bacteria are quantified in Figure 5B. The control samples (untreated bacteria) remained constant throughout the measurement period, exhibiting no significant changes in the live population. The addition of mast-MO at a concentration of 50 nM led to a decrease in the percentage of live bacteria after 4 h of incubation. This antibacterial effect becomes evident with increasing mast-MO concentration, resulting in a reduced bacterial viability of approximately 75% after incubation with 50 μM peptide. Additionally, bacterial aggregation is highlighted at the highest concentration of the peptide.

**Figure 5:**
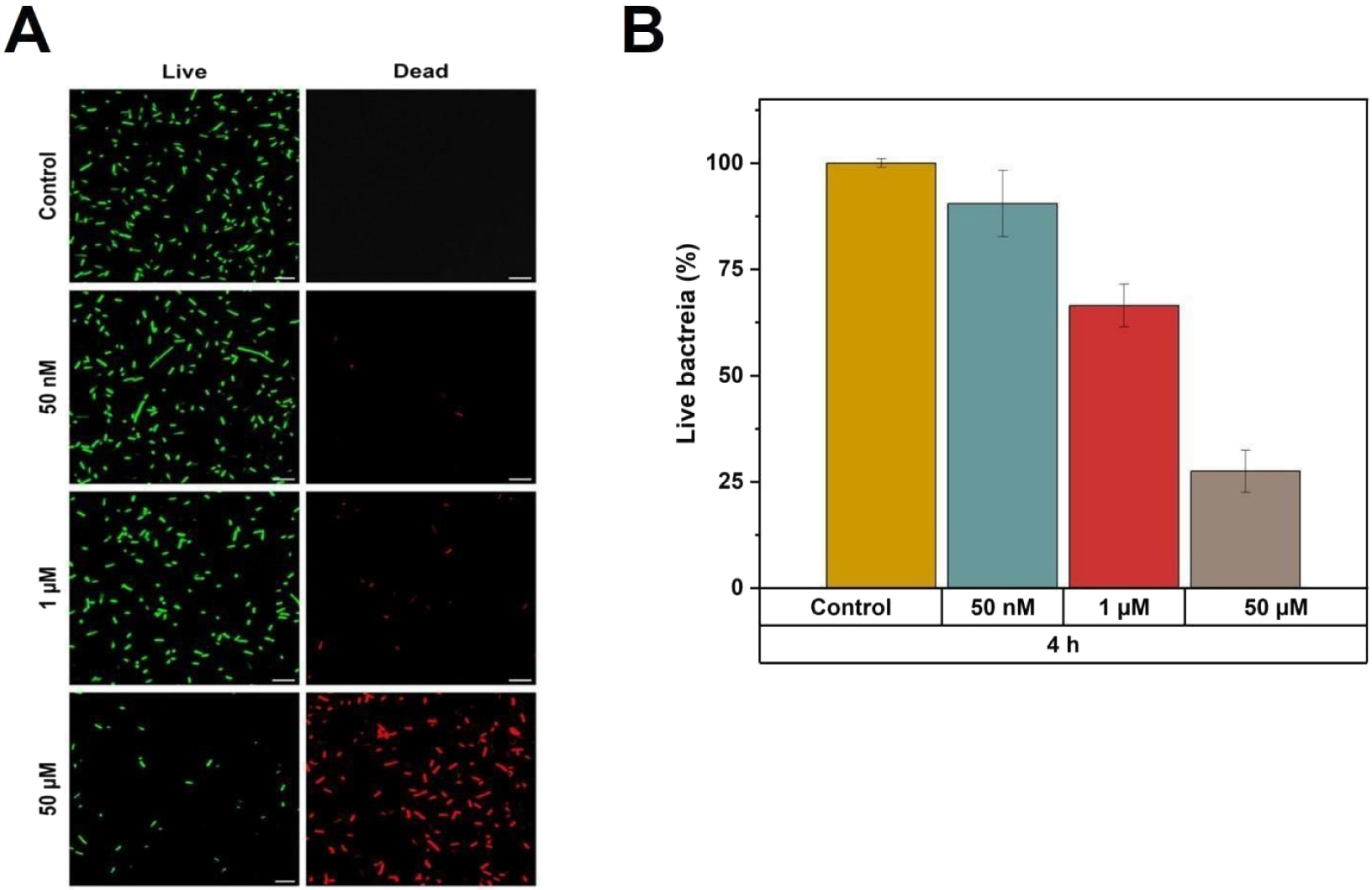
Confocal microscopy results using mast-MO, labeled or unlabeled, for 10^8^ CFU.mL^–1^ of *E. coli* in 10 mM Tris, pH 7.4, after treatment with different peptide concentrations. (**A**) Dead/live assay showed in green (SYTO 9, live) and red (PI, dead) for *E. coli* interacting with unlabeled mast-MO for 4 h. (**B**) Results of bacteria viability upon increasing peptide concentration as a function of untreated.

To get further insight into the localization of the peptide and the time scale of the cytotoxic effect, mast-MO-Cy5.5 was incubated with *E. coli* at 15 min, 1 h, and 4 h, as reported in Figure 6A. In this case, we could follow the fluorescence emitted by cyanine 5.5 attached to mast-MO during the experiment. Within the first 15 min of the experiment, the adsorption of mast-MO is fast, and the surface of the bacteria becomes decorated with the peptide, given the red fluorescence on the outer surface of the bacterial membrane. Interestingly, we observed a high degree of bacterial aggregation within the first 15 min (Figure 6B), and this effect becomes less evident with increasing incubation time. The percentage of mast-MO bound to the bacterial surface at different time points is quantified in Figure SI7. Some bacteria seem not to be decorated with mast-MO, as will be discussed in the following sections.

**Figure 6:**
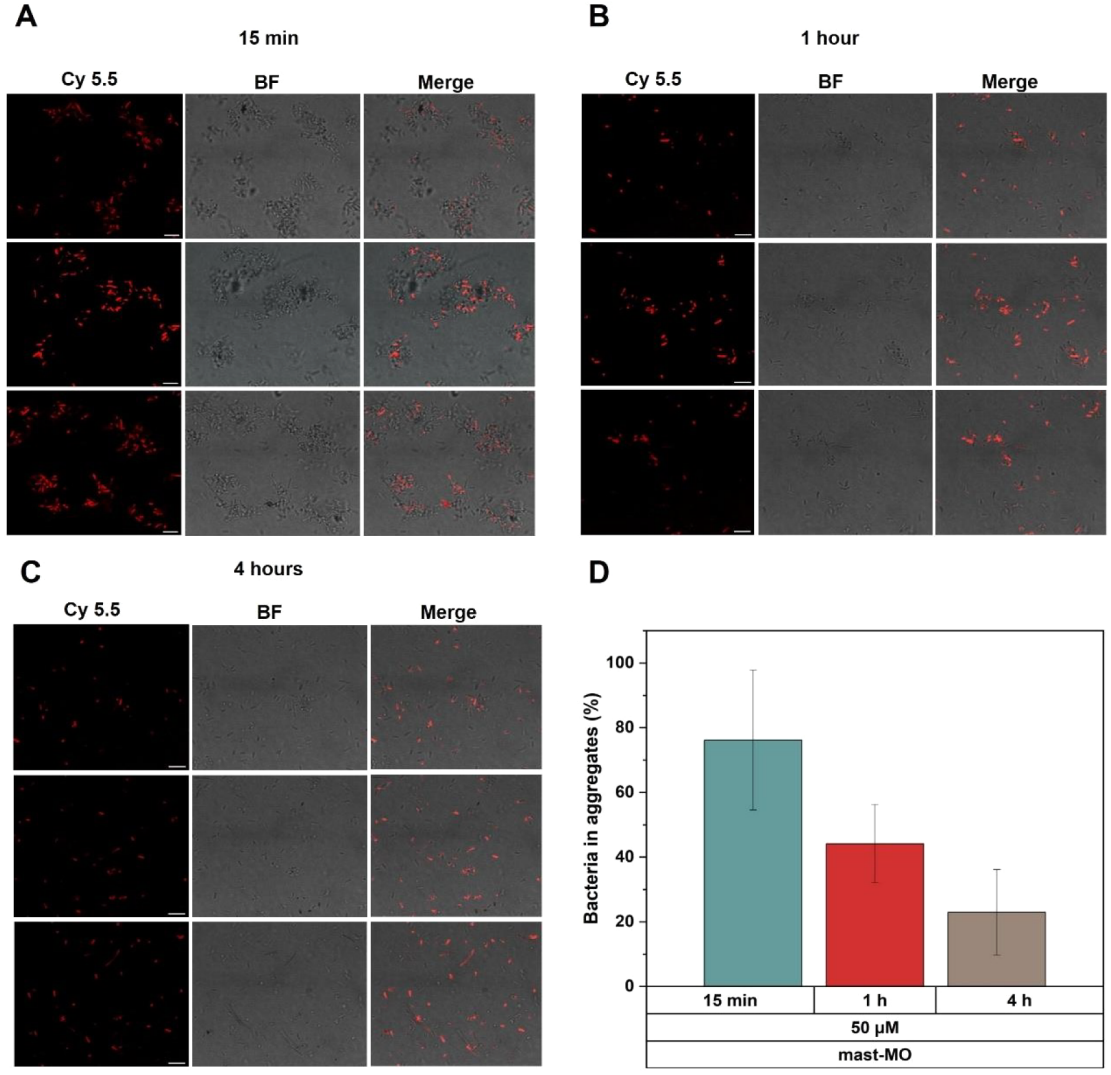
Confocal micrographs of *E. coli* at 10^8^ CFU mL^–1^ in 10 mM Tris, pH 7.4, after incubation with mast-MO-Cy5.5 for 15 min in **A**, 1 h in **B**, and 4 h in **C.** The images are reported as red channel (mast-MO-Cy5.5), bright field (BF), and merged channels (red+BF). **D** Quantification of bacteria aggregates presented in A, B and C at different incubation times and expressed as percentage.

## Discussion

### Mast-MO is Surface Active

This study aimed to investigate the mechanism of action for mast-MO to deepen our understanding of this peptide and its interactions with lipid membranes, either as SUVs or SLBs to mimic the bacterial membrane.^30,31^ Since the surface activity of this peptide can be linked to its *in vitro* activity, the molecular characterization of how mast-MO interacts with surfaces, including biomembrane interfaces, is pivotal.

The studies of peptide adsorption using QCM-D provide critical insights into the interactions of the AMP with either hydrophilic or hydrophobic surfaces. Mast-MO exhibited adsorption on both surfaces, including. This result suggests that the mast-MO exhibits surface activity. Given the primary structure, electrostatic and hydrophobic effects likely play a role in the adsorption to the bare surfaces, as previously shown for other AMPs.^32,33^

Mast-MO is predominantly composed of nonpolar amino acids.^17^ These residues may hinder the peptide’s adsorption onto hydrophilic surfaces at low peptide concentration. At 50 μM, mast-MO adsorbed more to hydrophilic surfaces than to hydrophobic ones, even after rinsing with buffer or water. Since the peptide is positively charged both in HBS and water (*pI* of 11.10), the salt concentration of the aqueous bulk mediates the adsorption by tuning the electrostatic interactions. On the hydrophobic surface, we observed some adsorption, albeit at a lower magnitude, due to attractive interactions between the hydrophobic residues of mast-MO and the surface. Overall, mast-MO thus behaves as an amphiphilic molecule. Other AMPs have been shown to exhibit amphiphilic surfactant-like behavior, a property intrinsically linked to their ability to disrupt lipid membranes. These peptides typically adopt amphipathic α-helical and other conformations,^34^ enabling insertion and destabilization of microbial membranes in a manner reminiscent of synthetic surfactants. Classic examples include melittin, which forms micelle-like aggregates and disrupts membranes through detergent-like action,^35^ and LL-37, which self-associates into supramolecular assemblies and exhibits surfactant behavior in epithelial barriers.^36^ Magainin 2 and cecropin A also display this property, forming helical structures that intercalate into membranes and induce curvature stress or pore formation.^37,38^ Even nonhelical peptides like indolicidin can interact with membranes in a surfactant-like manner due to their amphiphilic and aromatic nature.^39^ The surfactant-like behavior is central to their antimicrobial activity and suggests potential for broader biotechnological applications, including nanocarrier design and membrane remodeling.^40,41^

### The Effect of Mast-MO on Membranes is Concentration-Dependent

When an SLB composed of POPC: POPG 75:25 (mol/mol) is formed on a silica surface, and mast-MO is added to the system at various concentrations, the highest adsorption is observed at higher peptide concentrations. This can be linked to the favorable electrostatic interactions between the anionic POPG^42^ and the positively charged mast-MO in the studied solution conditions. At higher peptide concentrations, the adsorption to the SLB increases, leading to membrane disruption. In addition, the adsorption of the peptide onto the SLB results in the formation of a viscoelastic interfacial layer, indicating that the adsorbed layer is coupled with the surrounding aqueous solution. These properties of the membrane are also probed by the FRAP measurements with labeled membrane, where we saw a concentration-dependent effect of mast-MO in terms of diffusivity in the lipid membrane. At low peptide concentration, only a slight decrease in the diffusion coefficient of the labeled lipid can be observed. The highest mast-MO concentration led to complete disruption of the membrane organization at 50 μM. No fluorescence recovery was observed in the FRAP experiment, indicating that the lack of recovery reflects a complete loss of membrane integrity preventing lateral mobility and leading vesicle destabilization. Hence, the adsorption of the peptides to planar SLBs is associated with a concentration-dependent behavior (Figure 7B), as probed via both QCM-D and FRAP. Interestingly, the concentration-dependent effect of the peptide on the membrane is also seen when the peptide is added to SUVs in solution. The adsorption does not induce significant changes in size, ζ-potential, or morphology of the SUVs at low AMP concentration. When the ζ-potential is approaching neutrality, e.g., the amount of positively charged AMP balances the ζ-potential of the negatively charged vesicles, the first signs of deformation are observed at cryo-TEM. The presence of multilamellar and multivesicular structures at intermediate concentrations (1 μM) suggests that mast-MO can induce complex membrane reorganization before causing complete disruption. Increasing the concentration of mast-MO leads to more deformation and disruption of the membranes, the latter being the primary outcome of adsorption to SLBs with identical composition. One speculation on this concentration-dependent effect may be related to a local change in the curvature of the vesicles due to mast-MO adsorption, as the SUVs’ shape is deformed into ellipsoids from sphere upon peptide binding (Figure 4). As shown in previous reports, proteins and peptides can interact with lipid membranes, inducing a local curvature of the bilayer, which becomes more pronounced the higher the concentration in the solution.^11,43^ This tipping point in concentration/structure may be the product of balancing between the disorder induced by the peptide and the lipid membrane ability to main its shape. After a threshold dependent on the lipid composition and the protein/peptide, such an interaction leads to the deformation of the shape of the vesicle due to the constraint imposed by the protein on the specific region of the bilayer. In addition to this, some AMPs can sense and preferentially bind to regions of high membrane curvature due to an increase in the interfacial hydrophobicity of the membrane.^44^ Häffner et al.^20^ observed surface charge reversal at specific peptide concentrations, suggesting that the peptides effectively neutralize the negative charge of the vesicle surfaces, which may be enhanced in regions of high curvature due to increased accessibility of the negative lipids onto the membrane. Indeed, more investigations would be needed to assess the curvature-sensing ability of the peptide.^45^ Moreover, arginine-rich peptides induced a stronger induction of negative membrane curvature. Lipid and protein sorting, coupled to the membrane structure, were used to explain the interplay between Gaussian and mean curvatures and provide a mechanistic basis for the initial membrane deformation events potentially involved in cell entry pathways.

**Figure 7:**
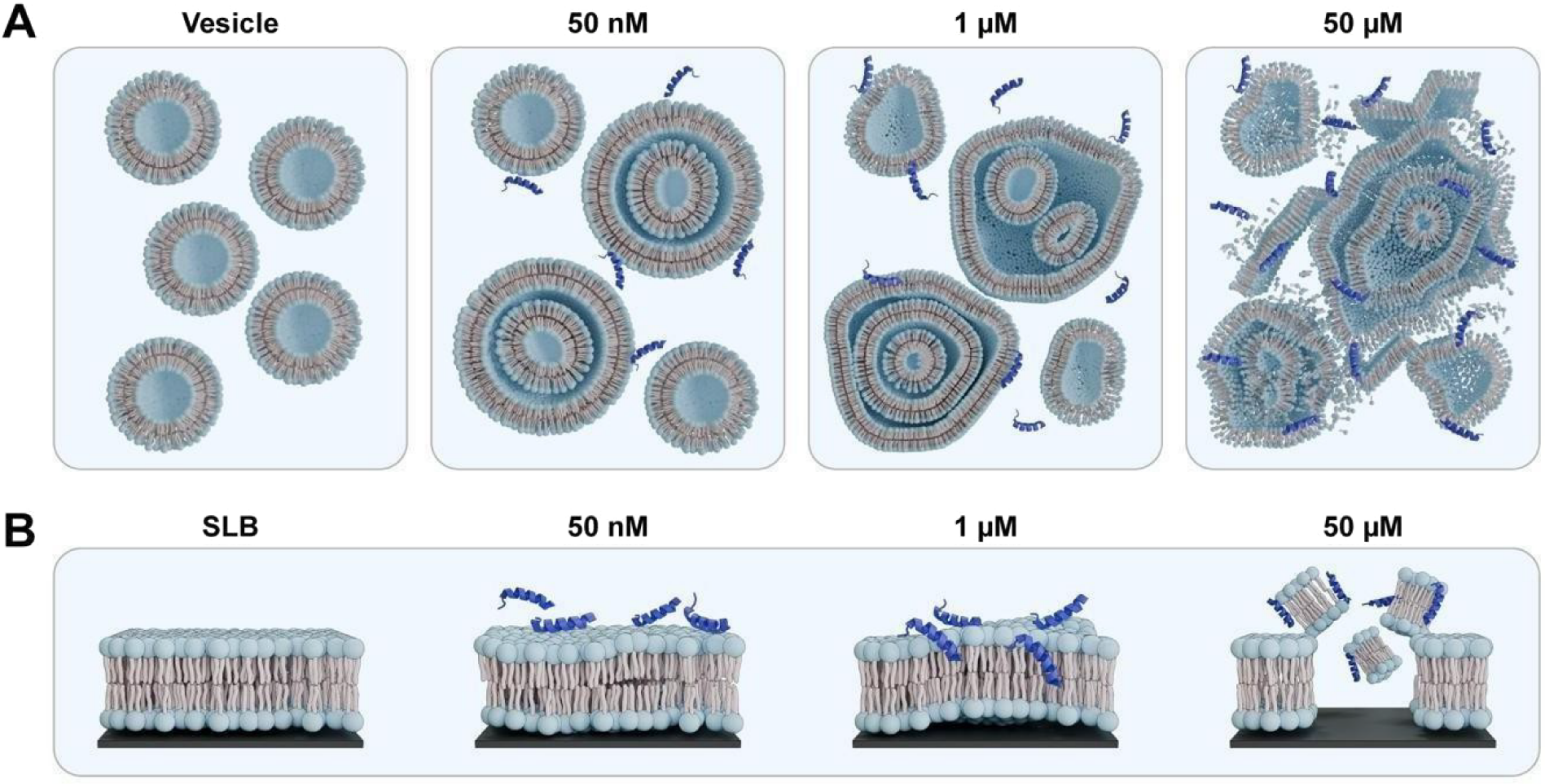
In panel **A,** schematic illustration showing morphological effect induced by different concentrations of mast-MO on SUVs. In panel **B,** structural changes induced by mast-MO adsorption to SLBs.

Indeed, further investigations on fully zwitterionic membranes or composed of lipids with different tail length may aid in drawing fundamental conclusions on the physicochemical behavior of mast-MO.

### Mast-MO Interacts with the Membrane via a Conformational Change

The results obtained by CD suggested that mast-MO adsorption onto SUVs is characterized by a transition from random coil to the α-helix upon increasing the lipid concentration. This behavior is similar to several other proteins^46^ and peptides^28,29^ adopt an α-helix as the most stable conformation at the interface of anionic membranes.

Surprisingly, the saturation point, e.g., the saturation of α-helical content, is reached at low lipid concentrations (i.e., low L:P ratios). This fact may be related to the size of the AMP, which is smaller than proteins such as α-synuclein, which require higher lipid concentrations to achieve this saturation. Therefore, more mast-MO molecules can be accommodated per vesicle, and fewer vesicles are needed to induce the complete conformational change of the peptide in solution. Peptides with stable α-helical structures have been shown to preferentially adsorb to curved membranes due to the increase of interfacial hydrophobicity of the membrane.^44,47^ The stable secondary structure observed in CD measurements may be related to findings reporting that amphipathic helices effectively may prefer and adsorb to curved membrane regions, enhancing their disruptive capability.^47,48^

### Influence of the Solution Conditions on Peptide Adsorption

HEPES buffer and pure water were also employed as media to highlight the effect of ionic strength on the AMP binding, in addition to HBS. In the studies using SLBs, lower amounts of mast-MO were found to adsorb to the membranes by changing from HBS to HEPES or water, as shown by the QCM-D results. The membranolytic effect of the peptide was found only in HBS and HEPES, but not when the experiment was conducted in water. These differences could be explained by the charged peptide in these environments. Pure water is usually more acidic than the buffers used in the study, meaning that the peptide is more positively charged when present in water than in the buffer, hence allowing for stronger electrostatic attraction to the anionic SLB. The HEPES buffer has a low ionic strength but the same pH as HBS. As the *pI* of the peptide should be the same in the two buffers, the presence of salt may aid in screening unfavorable peptide–peptide repulsion onto the membrane in the case of HBS; this adsorption then leads to the disruption of the SLB.

The desorption of the peptide was more pronounced when the rinsing solution was switched to water compared to the rinsing steps in HBS or HEPES. This preference may be explained by a change in the density and ionic content of the medium, which is coupled with the amount of mass adsorbed at the membrane interface, leading to a higher frequency shift. The adsorption of the peptide appears to be irreversible for all the solution conditions investigated, with approximately one-third of the peptide bound to the surfaces at the end of the experiment in all conditions, similar to findings from other peptide-surface interaction studies.^49^

Regarding the DLS data, an increase in hydrodynamic diameter is observed only in HBS, not in the other media. However, the charge reversal is induced in all the media, with a similar concentration of mast-MO, slightly higher for the high pH and ionic strength conditions. This is not surprising, as the ions in the buffer may shield the electrostatic field of the Stern layer, necessitating a higher peptide concentration to screen the charge of the anionic vesicles.

### Mast-MO Effects on Bacteria

The results obtained for the model systems are corroborated by the *in vitro* imaging of *E. coli* after incubation with mast-MO. The LIVE/DEAD essay yielded similar outcomes to those observed in the case of model membranes, specifically SUVs and SLBs, where aggregation and membrane disruptions were noted. These results are similar to the ones related to the concentration-dependent activity of LL-37 against *E. coli*.^50^ The confocal images revealed that mast-MO achieves maximum activity after just 15 min of incubation; this rapid action is highly desirable for antimicrobial agents and is consistent with other membrane-active AMPs. For instance, the synthetic peptide WLBU2 has been shown to cause significant membrane permeabilization in *P. aeruginosa* within 5 min of exposure.^51^ We also observed bacterial aggregation in addition to direct membrane disruption. After 4 h of incubation, approximately 25% of bacteria remained viable and they were aggregated in similar proportions (Figures 5B and 6B). However, we do not conclude that aggregation alone is the primary cause of cell death. Our data indicates that mast-MO exerts two complementary effects on *E. coli*: rapid membrane disruption leading to early loss of viability, detectable within 15 min of incubation (Figure 6), and induction of bacterial aggregation, likely arising from the exposure of hydrophobic membrane regions following peptide insertion in the membrane. The comparable percentages of living and aggregated bacteria after prolonged incubation suggest that these processes occur at the same time, but aggregation is not solely responsible for, bacterial death. This interpretation aligns with previous reports for other AMPs, such as LL-37, where both membrane permeabilization and bacterial aggregation contribute to the antimicrobial mechanism.^52^ As not all bacteria are decorated with mast-MO, a positive cooperative behavior may be connected to peptide adsorption; however, further investigations are needed to assess the cooperative nature of mast-MO adsorption to membranes fully.

### Considerations for the Mechanism of Action of Mast-MO

The physicochemical characterization provided for mast-MO can hint at its possible mechanism of action. In general, it is evident that the peptide is surface-active: it binds to bare surfaces (both hydrophilic and hydrophobic), and it destabilizes lipid packing, leading to the subsequent disruption of the bilayer at high concentrations (50 μM), which results in lipid removal from the membrane and subsequent aggregation in solution. This phenomenon is confirmed in both model systems and living bacteria, where clusters of lipids and mast-MO are observed.

Based on the multitechnique characterization, it is suggested that mast-MO interacts with the surface of the lipid bilayer, exhibiting a membranolytic effect, rather than translocating across the bilayer and inducing a lytic effect. The membranolytic effect is indeed associated with a threshold in terms of peptide concentration. Below this threshold, the reduction in the lateral diffusion coefficient is a sign of a strong interaction between the peptide and the membrane, which can lead to the biological activity of mast-MO. Bacteria death is already induced at 50 nM, at which the reduction of lateral diffusion occurs in model systems. This represents an essential key point as lateral mobility (0.04–3.5 μm^2^/s) in plasma membranes is fundamental for the correct function of living cells.^53,54^ Similar behavior has been reported for antimicrobial peptides such as LL-37 and melittin, which adsorb to the membrane surface, destabilize lipid packing, and induce aggregation or leakage in a concentration-dependent manner, without fully translocating across the bilayer.^55,56^ In the context of the literature on antimicrobials, many AMPs lead to membrane destabilization by inducing lipid-packing defects upon binding.^44,57,58^

## Conclusions

This study comprehensively investigates the interactions between an antimicrobial peptide, mast-MO, and lipid membranes, contributing to understanding its potential as an anti-microbial agent. These results provide an overview of the mode of action of mast-MO in model and living systems, corroborating the molecular steps involved in its mechanism of action. In addition, by leveraging complementary techniques, we highlighted small changes in dynamics and lateral organization of the membrane upon peptide adsorption. In conclusion, mast-MO primarily functions as a surface-active antimicrobial peptide destabilizing lipid membranes. At low concentrations, mast-MO initiates viscoelastic changes at the membrane and reduces lateral lipid diffusion, thereby affecting membrane dynamics. As the peptide concentration increases, it promotes lipid domain segregation, and membrane curvature is promoted, ultimately leading to membrane aggregation. These effects mimic those of amphiphilic surfactants. *In vitro* assays confirm the rapid adsorption of peptide to *E. coli*, resulting in bacterial aggregation and cell death within minutes, without intracellular penetration. This behavior positions mast-MO as a membrane-active peptide that leverages surface disruption rather than pore formation or translocation, representing a potent candidate for next-generation antimicrobial strategies.

Rather than penetrating the membrane to form stable pores, mast-MO works through surfactant-like surface adsorption, distorting lipid structure in a manner that induces curvature stress, destabilization, and lipid detachment/aggregation. This mechanism diverges from pore-forming peptides (e.g., melittin;^59^ α-helical AMPs^60^), it aligns with membrane-perturbing agents like indolicidin (which induces nonpore-forming membrane thinning via tryptophan-rich motifs^61^ and bombinin H2 (known for curvature-dependent bilayer disruption in anionic membranes^62^). Additionally, mast-MO operates without cytosolic translocation, correlating directly with biophysical alterations in membrane diffusivity and integrity^63^

Overall, mast-MO represents a promising candidate for developing next-generation antimicrobial agents, leveraging its unique mode of action to overcome existing limitations in treating bacterial infections.

## Experimental Section

### Materials

Mast-MO (>95% purity) was obtained from Thermo Fisher Scientific (USA). Mast-MO labeled with cyanine5.5 (mast-MO-Cy5.5) was sourced from ChinaPeptides Co., Ltd. (99% labeling efficiency). The lipids palmitoyloleoylphosphocholine (POPC) and palmitoyloleoylphosphoglycerol (POPG) (both with >99% purity) were purchased from Avanti Polar Lipids (Alabaster, USA). The BacLight Bacterial Viability Kit was acquired from Thermo Fisher Scientific Inc. (Waltham, USA). Lennox broth (LB Broth) was obtained from Sigma Aldrich (St. Louis, USA). Sodium chloride (≥99.0% purity), calcium chloride dihydrate (≥99.0% purity), and HEPES (-(2-Hydroxyethyl) piperazine-1-ethanesulfonic acid, *N*-(2-Hydroxyethyl) piperazine-*N*′-(2-ethanesulfonic acid)) (≥99.0% purity) were also purchased from Sigma-Aldrich. All chemicals used were of analytical grade and used without further purification.

### Lipid Membrane Preparation

Small unilamellar vesicles (SUVs) were prepared via the thin-layer evaporation method by aliquoting the desired amount of stock solution of POPC or POPG in chloroform (10 mg/mL), keeping the molar ratio between the two components fixed at 75:25, and reaching a total lipid concentration of 0.5 mg mL^–1^. After using an N2 stream to remove the organic solvent, the resulting lipid film was hydrated in the desired medium: HEPES buffer saline (HBS), HEPES, or water. The mixture was then vortexed 5 times to ensure the dispersion of the lipid films, obtaining a homogeneous milky solution that was then extruded 21 times through a 100 nm polycarbonate filter mounted in a LipoFast mini extruder (Avastin, Ottawa, Canada). Samples for quartz crystal microbalance with dissipation monitoring (QCM-D) and total internal reflection fluorescence (TIRF) microscopy experiment were instead sonicated in an ice water bath using a tip sonicator 0.5, amplitude 100% for 10 min (UP50H -Hielscher). Vesicles containing DHPE lipid labeled with Oregon Green (DHPE-OG) at a final concentration of 0.5 mol % in addition to POPC and POPG were used for the fluorescence microscopy experiments.

### Quartz Crystal Microbalance with Dissipation Monitoring to Study Peptide Adsorption

QCM-D was performed using a Q-Sense E4 system (Biolin Scientific, Gothenburg, Sweden) with four measurement cells, featuring SiO_2_ quartz crystals (QSensor, QSX303) with a fundamental frequency of (4.95 ± 0.05) MHz, a diameter of 14 mm, a thickness of 0.3 mm, and a mass sensitivity factor of 17.7 ng/cm^2^. Before each experiment, the crystals were cleaned using the protocol reported. Briefly, each sensor was washed for 5 min in a sonication bath at room temperature in 2% V/V Hellmanexx, followed by water and ethanol, and then dried with N2. After plasma cleaning in a vacuum for 10 min, the crystals were immediately introduced into the measurement cells to avoid contamination and dust adsorption. During the experiments, a flow rate of 0.1 mL/min was controlled by a peristaltic pump (Ismatec IPC 4-channel, Cole-Parmer GmbH, Germany) and a constant temperature of 25 °C was maintained. Stable baselines in water for frequency (*Δf*) and dissipation *(ΔD*) shifts were then recorded. For measurements with mast-MO alone, the desired medium was flushed in before peptide injection. In contrast, the deposition of the lipid bilayer was preceded by a flush of a solution of 4 mM CaCl_2_ for 10 min to ensure fusion and deposition of the vesicles. In this latter case, supported lipid bilayer (SLB) formation was observed when Δ*f* reached −23 Hz.^20^ The excess of vesicles in the cell was then washed with the desired buffer. After this, the desired concentrations of mast-MO were added sequentially (with a 10 min injection time), followed by a waiting period until the signals were stabilized. For the rinsing steps, the cells were washed with the desired buffer for approximately 1 h and then with water for 2 h. These experiments were repeated in triplicate, and the overtones seventh, ninth, and 11th of the frequency and dissipation were then exported for analysis. The data for the adsorption of mast-MO to SLBs were then normalized by subtracting the equilibrium values of *Δf* and *ΔD* from those after the injection of the peptide at different subsequent concentrations.

### Fluorescence Microscopy to Study Lateral Mobility of the Membrane

Experiments based on fluorescently labeled samples (both the peptide and the SLBs) were performed via total internal reflection fluorescence (TIRF) microscopy on a Nikon Eclipse TE2000-U microscope, using a Hamamatsu ORCA-Flash4.0 LT Digital scientific CMOS camera (C1140–22U) and a Nikon Apo TIRF 60× magnification oil-immersion objective to minimize the contribution of the bulk solution. The SLBs containing the lipid dye DHPE-OG488 and mast-MO-Cy5.5 were illuminated with Cobolt MLD compact diode lasers at 488 nm (30 mW) and 638 nm (30 mW), respectively. Images were recorded after SLB formation, mast-MO incubation, and rinsing in both channels to observe the effect of peptide binding to the bilayer. The lateral diffusion within the SLB was evaluated using fluorescence recovery after photobleaching (FRAP), before and after mast-MO addition to ensure the quality of the bilayer in terms of diffusion coefficient, D, and immobile fraction, γo. These parameters were evaluated according to a previously published method:^21^ a small region of the SLB was photobleached by focusing the laser for 5 s, and the recovery profile of the fluorescence was measured every 2 s for 2 min (60 images in total), as the final acquisition time. The recovery profiles were then analyzed using MATLAB and a prewritten script.^21^

### Size and Zeta Potential Determinations

The apparent hydrodynamic diameter (Dh) and zeta potential (ζ) of the SUVs (either POPC alone or POPC: POPG 75:25) were measured at 173° by dynamic light scattering (DLS) and electrophoretic light scattering (ELS), respectively, using a Zetasizer Nano ZS (Malvern Instruments, Malvern, UK) before and after peptide incubation. The samples were prepared by diluting the vesicles 1:50 in the desired medium to avoid multiple scattering and incubating mast-MO with the vesicles at different concentrations (from 50 pM to 250 μM) at 25 °C for 15 min. A second-order cumulant analysis was used to determine the apparent hydrodynamic diameter of the vesicles before and after peptide incubation. ζ potential values corresponding to the same time and concentration points were evaluated using the Helmholtz–Smoluchowski approximation after determining the electrophoretic mobility. In addition, the effect of different incubation times was studied using the same apparatus by incubating both types of vesicle compositions with mast-MO for 15 min, 1, 4, 8, and 24 h. All measurements were reported as triplicate (±standard deviation, SD) at 25 °C.

### Circular Dichroism to Evaluate AMP Conformational Changes

Circular dichroism (CD) spectroscopy was employed to evaluate the transition from random coil (unbound) to alpha-helix (bound) states. Mast-MO was mixed with the POPC: POPG vesicles in water, keeping the peptide concentration fixed at 5 μM and varying the lipid-to-peptide concentration (L/P) from 1 to 200. The CD signal in water was measured as a background in 1 mm path length quartz cuvettes (110-QS; Hellma et al.) using a JASCO (Tokyo, Japan) J-715 CD instrument, working with 20 nm/min scanning speed and 2 s response time, 1 nm bandwidth accumulated ten times. After this, samples for the peptide alone or the mixed peptide-SUVs at a different L:P were recorded under the same experimental conditions by accumulating the spectra four times. The measurements were repeated as triplicates with independent samples at 25 °C. The mean residual ellipticity (*MRE*) obtained for each sample was then calculated after subtracting the background (water) and normalized using the following equation:

1 $$\mathrm{MRE}=\left(\theta \times M\right)/\left(10^{*}l^{*}c\right)$$

where c is the mast-MO concentration, *M* is its molecular weight (2.06 kDa), *l* is the path length, and θ the raw CD signal. Ten is a conversion factor used to convert to the desired measurement units.

### Cryogenic Transmission Electron Microscopy to Study Vesicle Morphology

Cryogenic transmission electron microscopy (cryo-TEM) measurements were performed for vesicles alone and for vesicles incubated with different concentrations of mast-MO, using a JEM-2200FS microscope. The samples were diluted 1:50 prior to measurement to achieve the same lipid concentration used in the DLS and ELS experiments. The sample was prepared for imaging by pipetting a small volume (∼4 μL) on a holey carbon grid. It was then blotted at 25 °C in a controlled environment chamber and rapidly frozen by plunging the grid into liquid ethane (−183 °C). The grids were stored in liquid ethane until each microscopy session. Samples of vesicles and vesicles with mast-MO at different concentrations (50 nM, 1 μM, and 50 μM) were then imaged using a highly focused beam of electrons (λ ≈ 0.02 Å at 300 kV), with sample electron density contrasting. The temperature is maintained at ca. – 175 °C.

### Bacterial Viability Assay

*E. coli* ATCC25922 were grown to stationary phase in 25 mL Lennox broth (LB Broth; Sigma-Aldrich, St. Luis, USA) overnight at room temperature under shaking at 180 rpm. The bacteria were pelleted and washed by centrifugation (10,000*g*, 10 min, repeated twice) and then resuspended in 10 mM Tris pH 7.4. The samples were diluted in buffer to obtain an OD600 of 0.6, corresponding to 6 × 10^8^ CFU of *E. coli* per mL. Then, different concentrations of mast-MO (50 nM, 1 μM, and 50 μM) were added to 500 μL of bacteria suspension and incubated for 4 h at room temperature. This was followed by 10 min staining of 200 μL of the sample with 0.5 μL of a 1:1 (v/v) mixture of the fluorescent probes SYTO 9 (excitation/emission maxima 480/500 nm) and propidium iodide (590/635 nm).^22^ Subsequently, samples were imaged on an inverted confocal laser scanning microscope Leica DMI6000 with an SP5 tandem scanner operating in resonant mode with a 100× (1.4 NA) oil immersion objective. Bacteria (either alone or incubated with mast-MO) were plated onto a cover slide at 10^8^ CFU/mL^–1^, and then ten randomized, wide-field images (100 μm × 100 μm) were collected for each sample. The dead and alive bacteria fractions were quantified using ImageJ (National Institutes of Health, Bethesda, USA).^23,24^ Experiments were performed in triplicate at 25 °C.

### Confocal Laser Scanning Microscopy to Study the Localization of the AMP

Mast-MO-Cy5.5 was incubated with *E. coli* at 10^8^ CFU.mL^1^ at different times (15 min, 1, and 4 h) at each peptide concentration to understand peptide localization on/within the bacterium and its effect over time. Another test was performed using mast-MO-Cy5.5 only in 50 μM incubated with *E. coli* at 10^8^ CFU·mL^–1^ at different times (15 min, 1 h, and 4 h) to understand peptide effect over time. Quantification of bacterial aggregation and peptide binding was performed using ImageJ by counting bacterial cells. All imaging used the same equipment and protocols described previously, except for illumination at 633 nm (excitation wavelength) to visualize mast-MO-Cy5.5.

## Supplementary Material



## Notes

A word file containing additional QCM-D, DLS, CD, and confocal microscopy experiments is included in the Supporting Information file (PDF)

## ⊥

Marco Fornasier is currently at the Division of Nanobiotechnology, KTH Royal Institute of Technology and SciLifeLab, Tomtebodavägen 23B, Solna, Sweden

## Abbreviations

AMPs: antimicrobial peptides
TIRF: total internal reflection fluorescence
QCM-D: quartz crystal microbalance with dissipation monitoring
DLS: dynamic light scattering
ELS: electrophoretic light scattering
cryoTEM: cryogenic transmission electron microscopy
SLBs: supported lipid bilayers
SUVs: small unilamellar vesicles
CD: circular dichroism

## References

[1] Hunt D., Kates O. S. (2024). A brief history of antimicrobial resistance. AMA J. Ethics.

[2] Li G., Lai Z., Shan A. (2023). Advances of antimicrobial peptide-
based biomaterials
for the treatment of bacterial infections. Adv.
Sci..

[3] Lohan S., Mandal D., Choi W., Konshina A. G., Tiwari R. K., Efremov R. G., Maslennikov I., Parang K. (2022). Small amphiphilic peptides:
Activity against a broad range of drug-resistant bacteria and structural
insight into membranolytic proper- ties. J.
Med. Chem..

[4] Chen Y., Ye Z., Zhen W., Zhang L., Min X., Wang Y., Liu F., Su M. (2023). Design and synthesis
of broad-spectrum antimi- crobial
amphiphilic peptidomimetics to combat drug-re- sistance. Bioorg. Chem..

[5] Malanovic N., Lohner K. (2016). Antimicrobial peptides
targeting gram-positive bacteria. Pharmaceuticals.

[6] Duque H. M., Rodrigues G., Santos L. S., Franco O. L. (2023). The bi- ological
role of charge distribution in linear antimicrobial peptides. Expert Opin. Drug Discovery.

[7] Nielsen J. E., Prévost S. F., Jenssen H., Lund R. (2021). Impact of an- timicrobial
peptides on E. coli-mimicking lipid model mem- branes: Correlating
structural and dynamic effects using scat- tering methods. Faraday Discuss..

[8] Carrer M., Nielsen J. E., Cezar H. M., Lund R., Cascella M., Soares T. A. (2023). Accelerating lipid flip-flop at low concentrations:
A general mechanism for membrane binding peptides. J. Phys. Chem. Lett..

[9] Liao J., Yeong V., Obermeyer A. C. (2024). Charge-patterned
disor- dered peptides
Tune Intracellular phase separation in bacte- ria. ACS Synth. Biol..

[10] Baranova A. A., Alferova V. A., Korshun V. A., Tyurin A. P. (2025). Imaging-based profiling
for elucidation of antibacterial mechanisms of action. Biotechnol. Appl. Biochem..

[11] Cardoso M. H., de la Fuente-Nunez C., Santos N. C., Zasloff M. A., Franco O. L. (2024). NS-T in,
2024 undefined. Influence of antimicrobial peptides on the bacterial
membrane curvature and vice versa. Trends Microbiol..

[12] Zhang C., Yang M. (2022). Antimicrobial peptides:
From design to clinical application. Antibiotics.

[13] Torres M. D. T., Sothiselvam S., Lu T. K., de la Fuente- Nunez C. (2019). Peptide design
principles for antimicrobial applications. J.
Mol. Biol..

[14] Oshiro K. G. N., Freitas C. D. P., Rezende S. B., Orozco R. M. Q., Chan L. Y., Lawrence N., Lião L. M., Macedo M. L. R., Craik D. J., Cardoso M. H. (2024). Deciphering
the structure and mechanism of action of computer-designed mastoparan
peptides. FEBS J..

[15] Martell E. M., González-Garcia M., Ständker L., Otero-González A. J. (2021). Host defense peptides
as immunomodu-
lators: The other side of the coin. Peptides.

[16] Dos
Santos G. G., Bachi A. L. L., Rangel S. C., da Silva
Nali L. H., Daca T. S. L., Do Amaral J. B., Juliano Y., Natrielli-Filho D. G., Rossi F. E., Gil S. (2025). Acute and chronic response of supervised band-elastic resistance
exercise in systemic cy- tokines levels of bipolar disorders and schizophrenia
individ- uals: A pilot study. Behav. Brain Res..

[17] Silva O. N., Torres M. D. T., Cao J., Alves E. S. F., Rodrigues L. V., Resende J. M., Lião L. M., Porto W. F., Fensterseifer I. C. M., Lu T. K. (2020). Repurposing
a peptide toxin from wasp venom
into antiinfectives with dual antimicrobial and im- munomodulatory
properties. Proc. Natl. Acad. Sci. U. S. A..

[18] Vilas
Boas L. C. P., Buccini D. F., Berlanda R. L. A., Santos B. D. P. O., Maximiano M. R., Lião L. M., Gonçalves S., Santos N. C., Franco O. L. (2024). Antiviral activities of mastoparan-
L-derived peptides against human alphaherpesvirus 1. Viruses.

[19] Orozco R. M. Q., Oshiro K. G. N., Pinto I. B., Buccini D. F., Almeida C. V., Marin V. N., de Souza C. M., Macedo M. L. R., Cardoso M. H., Franco O. L. (2024). Employment of mastoparan-like
peptides to prevent Staphylococcus
aureus associated with bovine mastitis. J. Bacteriol..

[20] Häffner S. M., Parra-Ortiz E., Skoda M. W. A., Saerbeck T., Browning K. L., Malmsten M. (2021). Composition effects on photooxidative membrane destabilization
by TiO2 nanoparticles. J. Colloid Interface
Sci..

[21] Jönsson P., Jonsson M. P., Tegenfeldt J. O., Höök F. (2008). A method improving
the accuracy of fluorescence recovery after photobleaching analysis. Biophys. J..

[22] Boulos L., Prévost M., Barbeau B., Coallier J., Desjardins R. (1999). LIVE/DEAD
BacLight: Application of a new rapid staining method for direct enumeration
of viable and total bacteria in drinking water. J. Microbiol Methods.

[23] Collins T. J. (2007). ImageJ
for microscopy. BioTechniques.

[24] Schneider C. A., Rasband W. S., Eliceiri K. W. (2012). NIH Image
to ImageJ: 25 years of
image analysis. Nat. Methods.

[25] Bragazzi N. L., Amicizia D., Panatto D., Tramalloni D., Valle I., Gasparini R. (2015). Quartz-Crystal
Microbalance (QCM)
for public health: An overview of its applications. Adv. Protein Chem. Struct. Biol..

[26] Brown L., Wolf J. M., Prados-Rosales R., Casadevall A. (2015). Through the
wall: Extracellular vesicles in Gram-positive bacteria, mycobacteria
and fungi. Nat. Rev. Microbiol..

[27] Khavani M., Mehranfar A., Mofrad M. R. K. (2025). Antimicrobial peptide interactions
with bacterial cell membranes. J. Biomol. Struct.
Dyn..

[28] Silva O. N., Alves E. S., de la
Fuente-Núñez C., Ribeiro S. M., Mandal S. M., Gaspar D., Veiga A. S., Castanho M. A., Andrade C. A., Nascimento J. M. (2016). Structural studies of a lipid-binding peptide
from tunicate hemocytes
with anti-biofilm activity. Sci. Rep..

[29] Rios T. B., Maximiano M. R., Fernandes F. C., Amorim G. C., Porto W. F., Buccini D. F., Nieto Marín V., Feitosa G. C., Freitas C. D. P., Barra J. B. (2024). Anti-staphy peptides rationally designed
from Cry10Aa bacterial protein. ACS Omega.

[30] Hasan, M. ; Yamazaki, M. Elementary processes and mechanisms of interactions of antimicrobial peptides with membranes-single giant unilamellar vesicle studies Antimicrobial Peptides. Advances in Experimental Medicine and Biology Springer Singapore 2019 1117 17–32 10.1007/978-981-13-3588-4_3 30980351

[31] Sondhi P., Lingden D., Stine K. J. (2020). Structure,
formation, and biological
interactions of supported lipid bilayers (SLB) incorporating lipopolysaccharide. Coatings.

[32] Mehranfar A., Khavani M., Mofrad M. R. K. (2023). Adsorption process
of various antimicrobial
peptides on different surfaces of cellulose. ACS Appl. Bio Mater..

[33] Park P., Matsubara D. K., Barzotto D. R., Lima F. S., Chaimovich H., Marrink S. J., Cuccovia I. M. (2024). Vesicle protrusion induced by antimicrobial
peptides suggests common carpet mechanism for short antimicrobial
peptides. Sci. Rep..

[34] Serian M., Mason A. J., Lorenz C. D. (2024). Emergent
conformational and aggregation
properties of synergistic antimicrobial peptide combinations. Nanoscale.

[35] Tosteson M. T., Holmes S. J., Razin M., Tosteson D. C. (1985). Melittin lysis of
red cells. J. Membr. Biol..

[36] Oren Z., Lerman J. C., Gudmundsson G. H., Agerberth B., Shai Y. (1999). Structure and organization of the
human antimicrobial peptide LL-37
in phospholipid membranes: Relevance to the molecular basis for its
non-cell-selective activity. Biochem. J..

[37] Andreu D., Rivas L. (1998). Animal antimicrobial
peptides: An overview. Biopolymers.

[38] Matsuzaki K., Sugishita K. I., Harada M., Fujii N., Miyajima K. (1997). Interactions
of an antimicrobial peptide, magainin 2, with outer and inner membranes
of Gram- negative bacteria. Biochim. Biophys.
Acta, Biomembr..

[39] Rozek A., Friedrich C. L., Hancock R. E. (2000). Structure of the
bovine antimicrobial
peptide indolicidin bound to dodecylphosphocholine and sodium dodecyl
sulfate micelles. Biochemistry.

[40] Brogden K.
A. (2005). Antimicrobial
peptides: Pore formers or metabolic inhibitors in bacteria?. Nat. Rev. Microbiol..

[41] Chen C., Chen J., Yu Q., Zhang J., Niu X., Hao L., Yang L., Zhao Y. (2020). Effects of salts on the self-assembly
behavior and antibacterial activity of a surfactant-like peptide. Soft Matter.

[42] Rice A., Wereszczynski J. (2017). Probing the
disparate effects of arginine and lysine
residues on antimicrobial peptide/bilayer association. Biochim. Biophys. Acta, Biomembr..

[43] Makasewicz K., Linse S., Sparr E. (2024). Interplay
of α- synuclein with
lipid membranes: Cooperative adsorption, membrane remodeling and coaggregation. JACS Au.

[44] Pajtinka P., Vácha R. (2024). Amphipathic
helices can sense both positive and negative
curvatures of lipid membranes. J. Phys. Chem.
Lett..

[45] Baxová, K. L. ; Koikkara, J. ; Allolio, C. From molecular insight to mesoscale membrane remodeling: Curvature generation by arginine-rich cell-penetrating peptides. bioRxiv. 10.1101/2025.04.14.648709. PMC13103641 41873931

[46] Andersson A., Linse S., Sparr E., Fornasier M., Jönsson P. (2024). The density of anionic lipids modulates
the adsorption
of α- synuclein onto lipid membranes. Biophys. Chem..

[47] Liang Y., Zhang Y., Huang Y., Xu C., Chen J., Zhang X., Huang B., Gan Z., Dong X., Huang S., Li C., Jia S., Zhang P., Yuan Y., Zhang H., Wang Y., Yuan B., Bao Y., Xiao S., Xiong M. (2024). Helicity-directed recognition of
bacterial phospholipid via radially amphiphilic antimicrobial peptides. Sci. Adv..

[48] Nepal B., Leveritt J., Lazaridis T. (2018). Membrane curvature
sensing by amphipathic helices: Insights from implicit membrane modeling. Biophys. J..

[49] Yang Y., Huang J., Dornbusch D., Grundmeier G., Fahmy K., Keller A., Cheung D. L. (2022). Effect
of surface
hydrophobicity on the adsorption of a pilus-derived adhesin-like peptide. Langmuir.

[50] Benfield A. H., Henriques S. T. (2020). Mode-of-action
of antimicrobial peptides: Membrane
disruption vs. intracellular mechanisms. Front.
Med. Technol..

[51] Vasilchenko A. S., Rogozhin E. A. (2019). Sub-inhibitory effects
of antimicrobial peptides. Front. Microbiol..

[52] Wu C. L., Peng K. L., Yip B. S., Chih Y. H., Cheng J. W. (2021). Boosting
synergistic effects of short antimicrobial peptides with conventional
antibiotics against resistant bacteria. Front.
Microbiol..

[53] Weng J., Ren J. (2006). Luminescent quantum
dots: A very attractive and promising tool in
biomedicine. Curr. Med. Chem..

[54] Manz, A. ; Dittrich, P. S. ; Pamme, N. ; Iossifidis, D. Bioanalytical chemistry; World Scientific Publishing Company, 2015.

[55] Dürr U. H., Sudheendra U. S., Ramamoorthy A. (2006). LL-37, the only human member of the
cathelicidin family of antimicrobial peptides. Biochim. Biophys. Acta, Protein Struct. Mol. Enzymol..

[56] Raghuraman H., Chattopadhyay A. (2007). Melittin: A membrane-active peptide with diverse functions. Biosci. Rep..

[57] Larsen A. H. (2022). Molecular
dynamics simulations of curved lipid membranes. Int. J. Mol. Sci..

[58] Almeida P. F. (2023). In search
of a molecular view of peptide- lipid interactions in membranes. Langmuir.

[59] Zan B., Ulmschneider M. B., Ulmschneider J. P. (2025). The difference between MelP5 and
melittin membrane poration. Sci. Rep..

[60] Rahman F., Halder S., Rahman S., Hossen M. L. (2025). Investigating the
therapeutic ability of novel antimicrobial peptide dendropsophin 1
and its analogues through membrane disruption and monomeric pore formation. J. Phys. Chem. B.

[61] Nielsen J. E., Bjørnestad V. A., Lund R. (2018). Resolving the structural interactions
between antimicrobial peptides and lipid membranes using small-angle
scattering methods: The case of indolicidin. Soft Matter.

[62] Gagandeep K. R., Narasingappa R. B., Vyas G. V. (2024). Unveiling mechanisms of antimicrobial
peptide: Actions beyond the membranes disruption. Heliyon.

[63] Sun J., Yang F., Zheng Y., Huang C., Fan X., Yang L. (2024). Pathogenesis and interaction
of neutrophils and extracellular vesicles
in noncancer liver diseases. Int. Immunopharmacol..

