# Supplementary material for: A Bioinspired Mastoparan Exhibits Concentration-Dependent Anti-Bacterial Activity via Membrane Disruption
Source: ACS Appl Mater Interfaces. 2025 Nov 24;17(49):66235–48. doi: 10.1021/acsami.5c14290 (PMC12874362; doi:10.1021/acsami.5c14290)
Supplement: Supplementary file 1 [file am5c14290_si_001.pdf]

Supplementary Information

A Bioinspired Mastoparan Exhibits Concentration-Dependent Antibacterial Activity via Membrane Disruption

Gisele R. Rodrigues [‡,1,2,], Marco Fornasier [†,‡,2,*], Lucrezia Caselli [2], Martin Malmsten [2,4], Emma Sparr [2], Peter Jönsson [2], Octavio L. Franco [1,3,*]*

[1]Centro de Análises Proteômicas e Bioquímicas, Pós-Graduação em Ciências Genômicas e Biotecnologia, Universidade Católica de Brasília, DF, Brasília, 70790160, Brazil

[2] Dvision of Physical Chemistry, Lund University, SE-221 00 Lund, Sweden

[3] S-inova Biotech, Programa de Pós-Graduação em Biotecnologia, Universidade Católica Dom Bosco, MS, Campo Grande, 79117900, Brazil

[4] Department of Pharmacy, University of Copenhagen, DK-2100 Copenhagen, Denmark

[‡]These authors equally contributed to this work

[†] Marco Fornasier currently at Division of Nanobiotechnology, KTH Royal Insititute of Science and SciLifeLab, Tomtebodavä- gen 23B, Solna, Sweden.

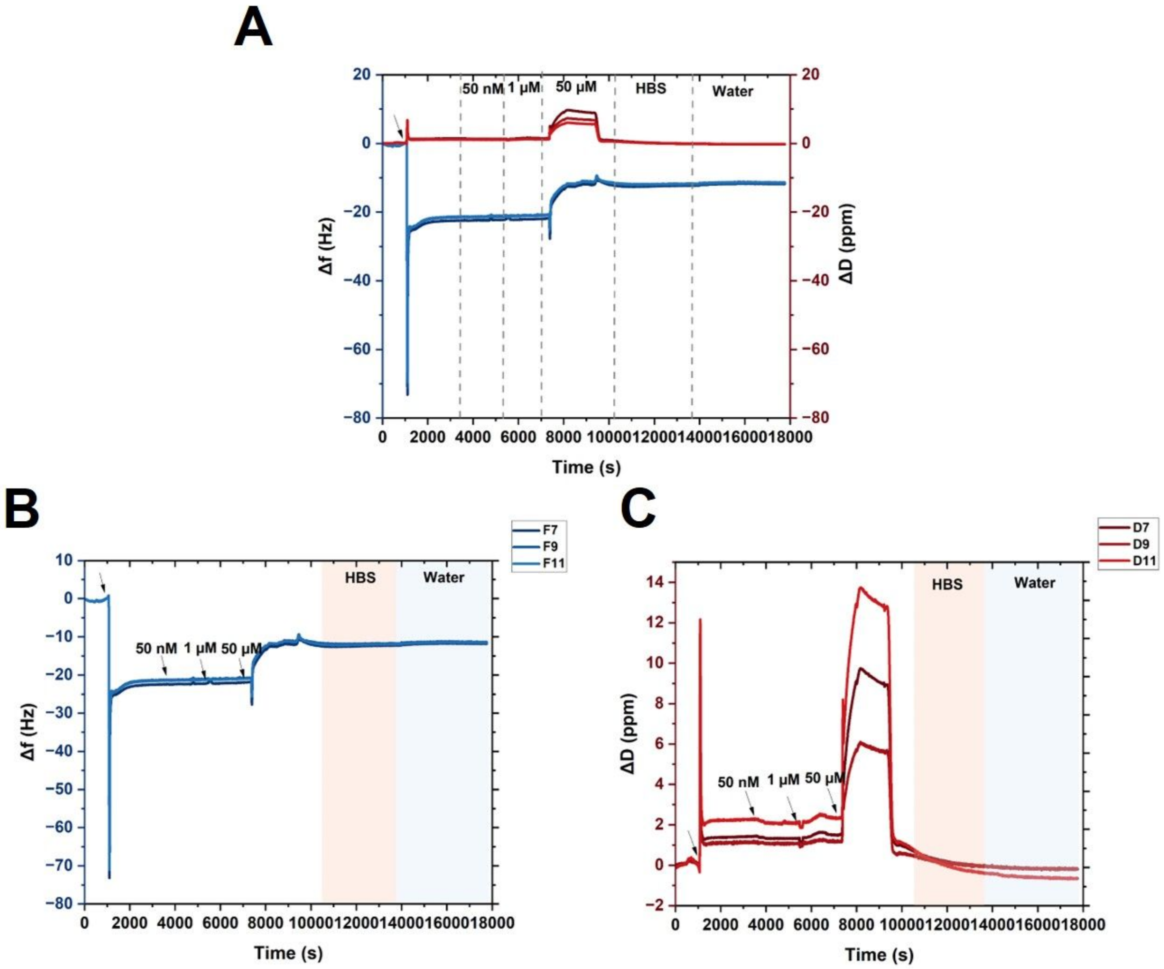

Figure S1. Full view of the QCM-D experiment for the POPC:POPG SLB in HEPES. The arrow points at the injection of the vesicles and the peptide was added only after stabilization of the frequency and dissipation signals.

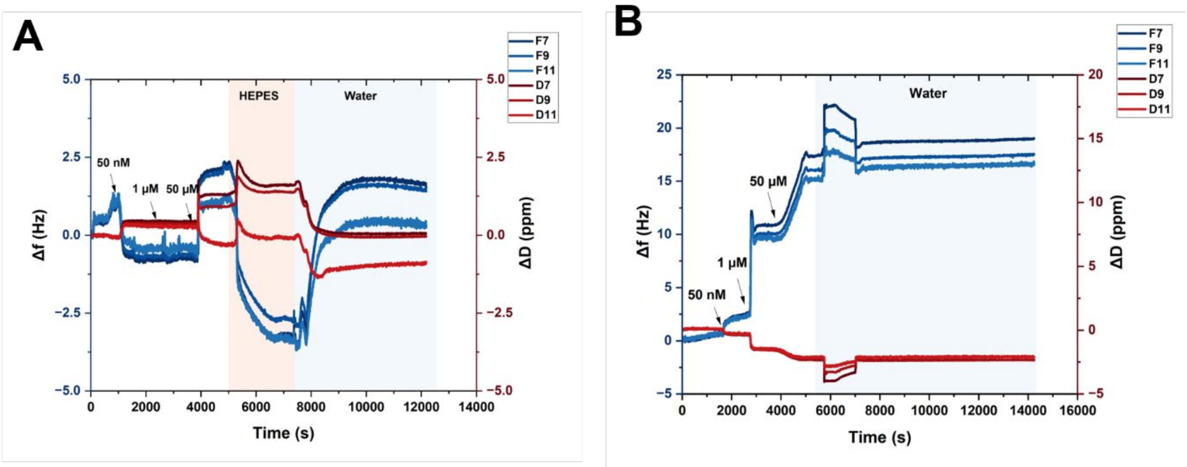

Figure S2. Representative QCM-D measurements on SLBs made of POPC:POPG bilayers with mast-MO in HEPES in A, and water in B as already shown in Figure 1C of the manuscript for the bilayers in HBS.

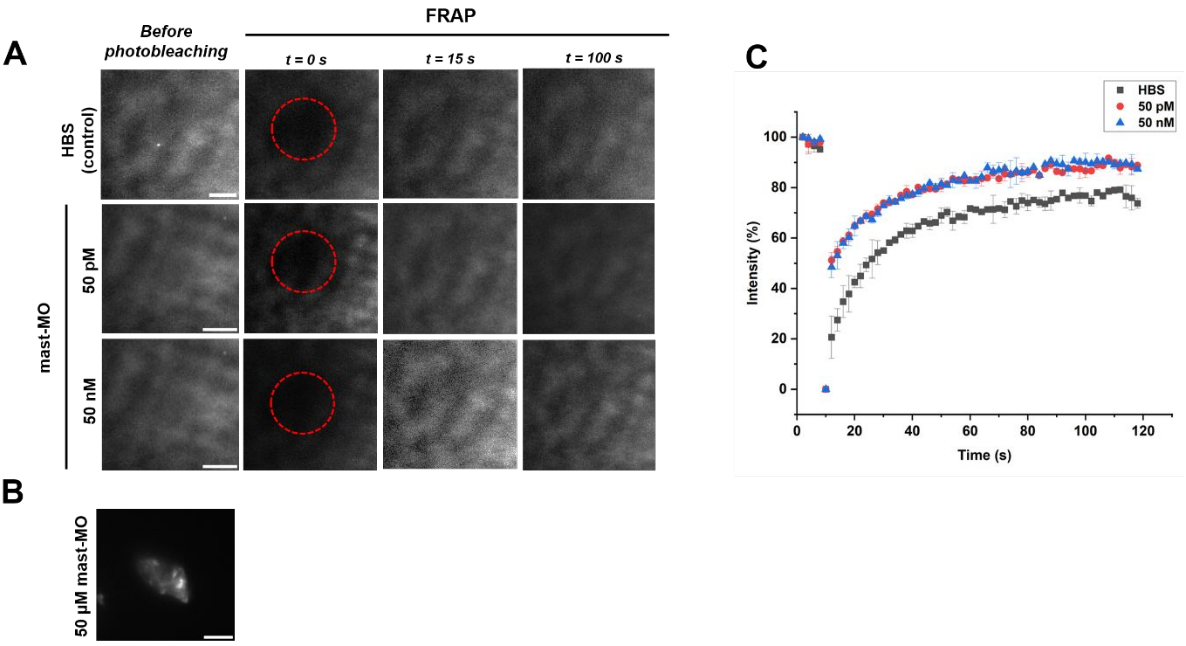

Figure S3. Representative FRAP images of DHPE-OG in POPC SLBs before and after different concentrations of mast-MO. The images represent the time points before bleaching and after the FRAP experiments by following the recovery in the dotted circle. (B) FRAP profile was obtained by evaluating the fluorescence intensity in each region of interest over time for the POPC SLB in HBS (black squares) after the addition of 50 pM (red circles) and 50 nM (blue triangles) of mast-MO.

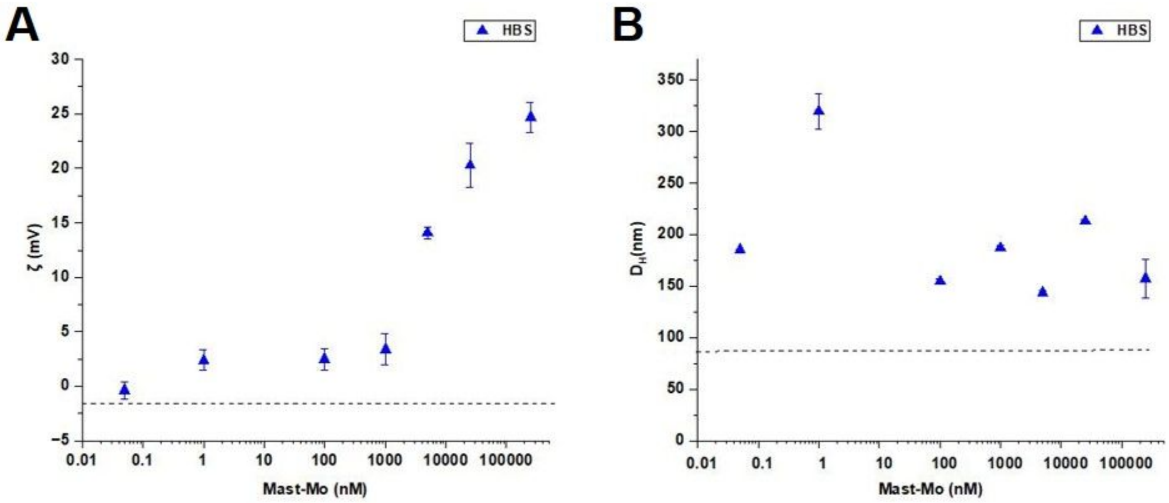

Figure S4. Average values of zeta potential in A and apparent hydrodynamic diameter in B of POPC vesicles incubated for 15 minutes with different concentrations of mast-MO at 25 °C. All data are reported as average values ± SD. The POPC:POPG vesicles zeta potential and hydrodynamic diameter (in the absence of peptide) are reported as a black dotted line in both plots as a control.

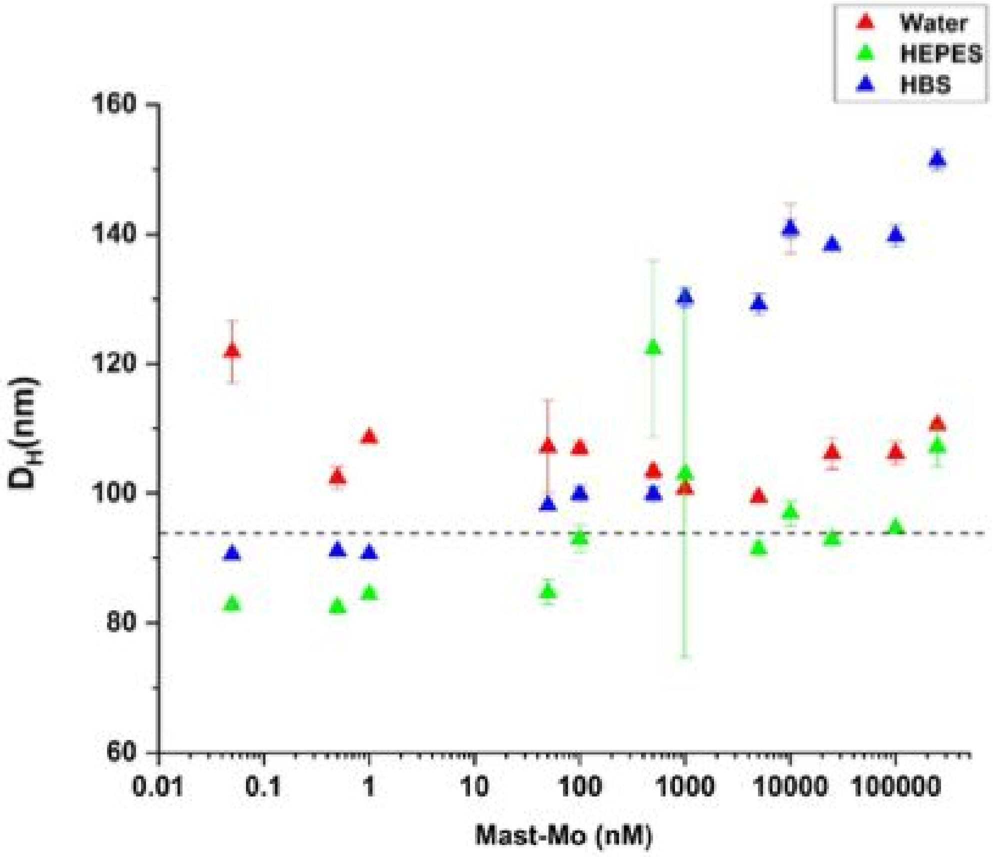

Figure S5. Trend of the apparent hydrodynamic diameter, D_h, of POPC:POPG vesicles incubated with different mast-MO concentrations in HBS, HEPES and water. This is the full trend reported in Figure 3B, without the colored dotted lines representing the average values of the trends. The mean apparent hydrodynamic diameter of the SUVs in the absence of peptide is still reported as black dotted line as a reference.

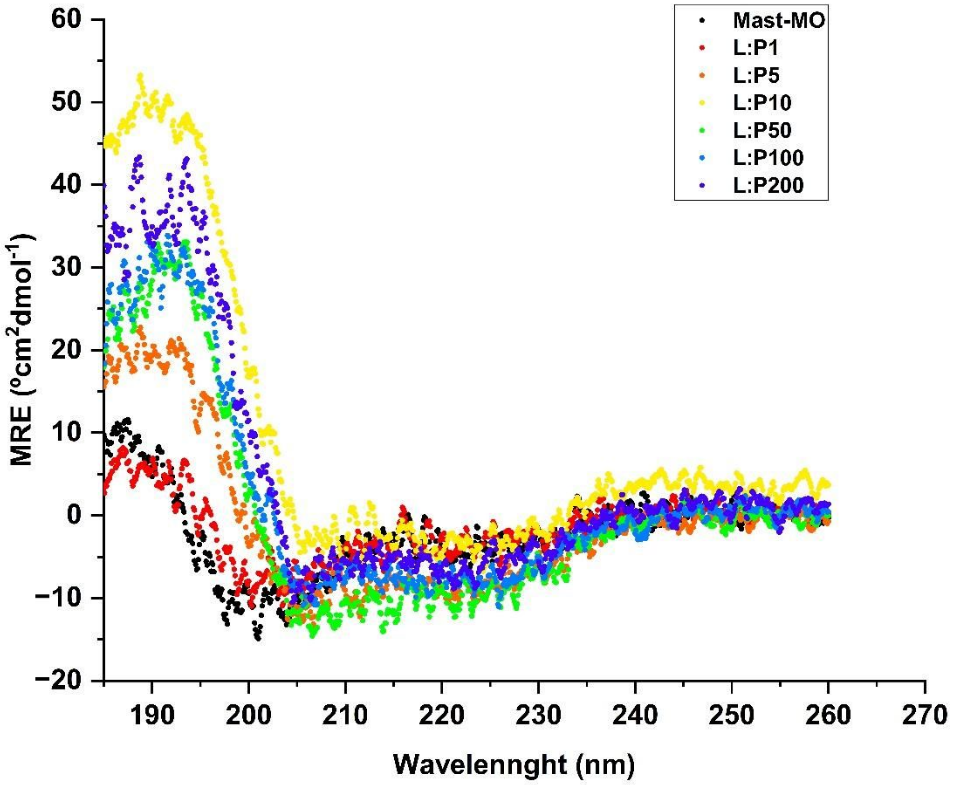

Figure S6. MRE values were evaluated by CD spectroscopy and through equation one for 5 μM mast-MO at different L:P ratios (SUVs composed by POPC: POPG, 75:25) as a function of wavelength in water.

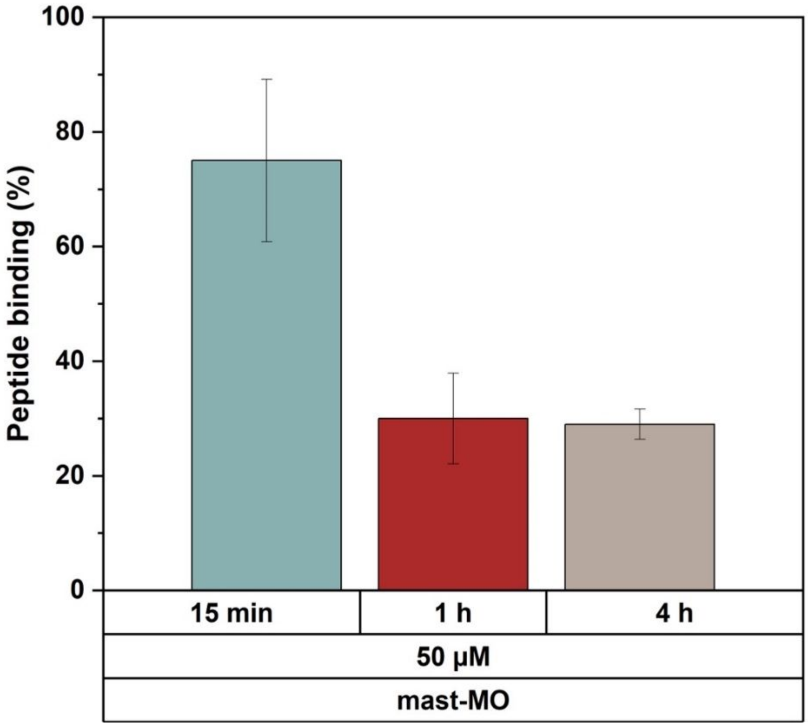

Figure S7. Confocal microscopy results using 50 µM labelled mast-MO, for 10^8 CFU/mL^{-1} of *E. coli* in 10 mM Tris pH 7.4, at different incubation time to verify the amount of peptide binding on *E. coli* over time.
